# Probabilistic Inference: Task Dependency and Individual Differences of Probability Weighting Revealed by Hierarchical Bayesian Modeling

**DOI:** 10.3389/fpsyg.2016.00755

**Published:** 2016-05-27

**Authors:** Moritz Boos, Caroline Seer, Florian Lange, Bruno Kopp

**Affiliations:** Department of Neurology, Hannover Medical SchoolHannover, Germany

**Keywords:** hierarchical Bayesian modeling, probabilistic inference, Bayesian inference, probability weighting, prospect theory

## Abstract

Cognitive determinants of probabilistic inference were examined using hierarchical Bayesian modeling techniques. A classic urn-ball paradigm served as experimental strategy, involving a factorial two (prior probabilities) by two (likelihoods) design. Five computational models of cognitive processes were compared with the observed behavior. Parameter-free Bayesian posterior probabilities and parameter-free base rate neglect provided inadequate models of probabilistic inference. The introduction of distorted subjective probabilities yielded more robust and generalizable results. A general class of (inverted) S-shaped probability weighting functions had been proposed; however, the possibility of large differences in probability distortions not only across experimental conditions, but also across individuals, seems critical for the model's success. It also seems advantageous to consider individual differences in parameters of probability weighting as being sampled from weakly informative prior distributions of individual parameter values. Thus, the results from hierarchical Bayesian modeling converge with previous results in revealing that probability weighting parameters show considerable task dependency and individual differences. Methodologically, this work exemplifies the usefulness of hierarchical Bayesian modeling techniques for cognitive psychology. Theoretically, human probabilistic inference might be best described as the application of individualized strategic policies for Bayesian belief revision.

## Introduction

Knight (1921) distinguished between risky worlds, referring to situations where perfect knowledge about probabilities is present and uncertain worlds, referring to situations where probabilities remain unknown. Savage (1954) made a similar distinction when he introduced the term *small worlds* for situations where all alternatives and their probabilities are known. In contrast, relevant information is unknown and/or must be estimated in *large worlds* (see also Johnson and Busemeyer, 2010; Volz and Gigerenzer, 2012).

**Figure 1:**
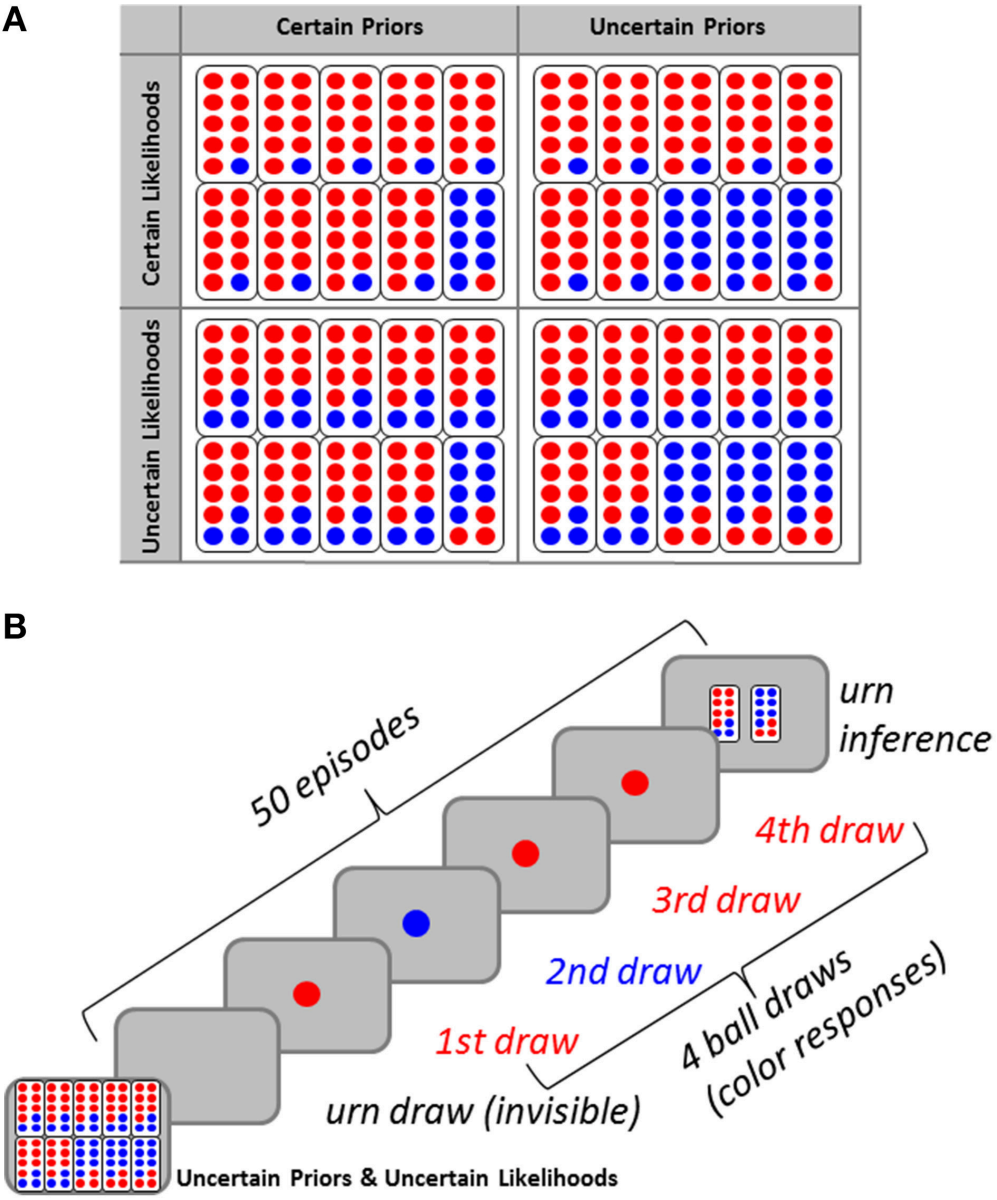
**(A)** Participants were graphically informed about the four probability conditions of the urn-ball task [certain priors (*P* = 0.9, *P* = 0.1), uncertain priors (*P* = 0.7, *P* = 0.3), certain likelihoods (*P* = 0.9, *P* = 0.1), uncertain likelihoods (*P* = 0.7, *P* = 0.3)]. **(B)** Each of the four probability conditions (here, the uncertain priors and uncertain likelihoods condition serves as an example) comprised 50 consecutive episodes of sampling that consisted of the invisible drawing of one urn, the visible drawing of a sample of four balls from that urn (sequential drawing with replacement). Participants indicated which urn they considered more likely, given the number of blue balls drawn (zero to four), and based on the condition (prior probability and likelihood function).

Small worlds provide opportunities for analysing Bayesian inference. In Bayesian decision theory (Jaynes, 2003; MacKay, 2003; Robert, 2007), degrees of belief in states of the world are specified. Bayesian inference updates prior beliefs using new evidence to derive posterior beliefs. Figure 1A shows small worlds, vested as an urn-ball task (Phillips and Edwards, 1966), consisting of two binary random variables, one representing unobservable states of the world (i.e., urns, *H* ∈ {*H*_1_, *H*_2_}), the other representing observable events (i.e., balls, *e* ∈ {0, 1}). Participants were asked for inferences about the current hidden state of that world, given small samples of events which could have been generated from either state (Figure 1B). To introduce experimental variance, we manipulated the task's probabilistic contingencies at two levels. First, we introduced uncertainty about the sort of urn containing balls (by sampling urns from two probability distributions). Second, we manipulated the proportion of ball colors within each urn. We will refer to these probabilistic contingencies as prior probabilities and likelihoods, respectively.

Thus, participants are informed about the world's stochastic structure, and they have access to evidence generated from one of these states. Participants represent knowledge-based and evidence-based degrees of belief in world states as probabilities. Specifically, participants hold informed prior beliefs [i.e., *P*(*H*_1_), *P*(*H*_2_) = 1 − *P*(*H*_1_)] and likelihoods [i.e., *P*(*E*|*H*_1_), *P*(*E*|*H*_2_)], i.e., conditional probabilities for some evidence, given each of the states. According to Bayes' theorem, prior probabilities are combined with likelihoods to provide posterior probabilities (Gold and Shadlen, 2007).

A long history of studies demonstrates that human judgment deviates from Bayesian decision theory (Kahneman et al., 1982). Initially, Edwards coined the term “conservatism” to describe probabilistic inference in which persons over-weigh prior beliefs (base rates) and under-weigh new sample evidence when compared to Bayesian decision theory (Edwards, 1982). Shanteau's work (1972, 1975) was centered around delineating conditions for the cognitive (sub-)additivity of new sample evidence. Later research identified the *base rate neglect* (Kahneman and Tversky, 1973; Bar-Hillel, 1980), i.e., a cognitive bias which indicates that the posterior probability of hypothesis *H*, given evidence *e*, is assessed without taking into account the prior probability (*base rate*) of *H*. Base rate neglect represents a particularly pertinent class of deviations in probabilistic judgment from Bayesian decision theory (Koehler, 1996).

**Figure 2:**
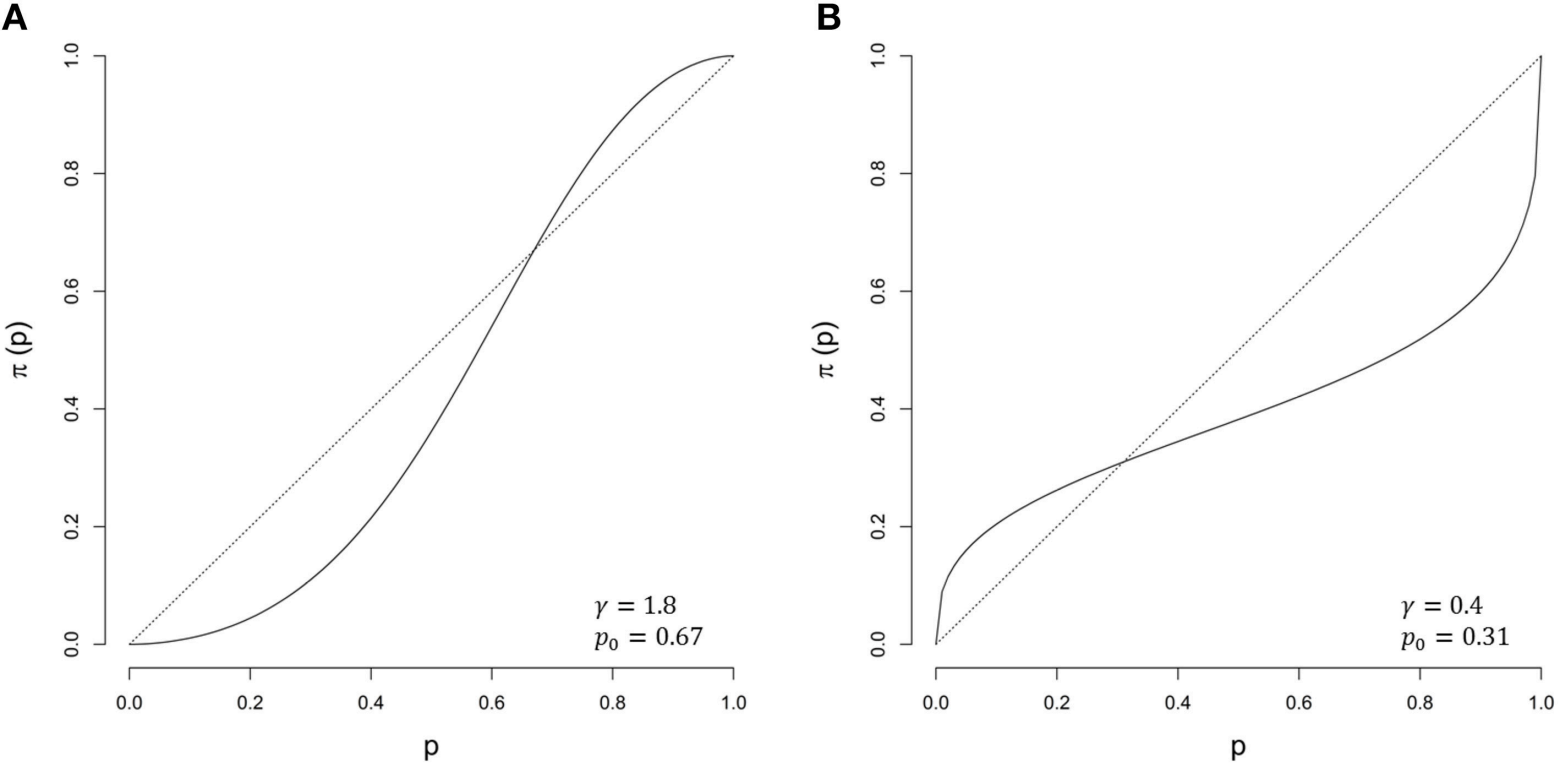
**Examples of probability weighting functions as proposed by Zhang and Maloney (2012). (A)** γ = 1.8 and *p*_0_ = 0.67 (solid) against the unweighted probabilities (dotted). **(B)** γ = 0.4 and *p*_0_ = 0.31 (solid) against the unweighted probabilities (dotted).

Prospect Theory (Kahneman and Tversky, 1979; Tversky and Kahneman, 1992) successfully describes economic decision behavior. Its probabilistic part proposed subjective probabilities used in decision-making to be non-linear functions of objective probabilities, with their relationships being best described by S-shaped probability weighting functions, leading to a tendency to overestimate low probabilities and to underestimate high probabilities (see Figure 2). One of our reviewers brought our attention to the fact that the notion of weighting probabilities was around 15 years prior to the introduction of Prospect Theory (Edwards, 1954), and that Edwards' early discussion of probability weighting is in the context of probabilistic inference rather than risky choice, which is the focus of Prospect Theory. Such S-shaped probability distortions are ubiquitous in research on probabilistic inference (Gonzalez and Wu, 1999; Luce, 2000; Zhang and Maloney, 2012; Cavagnaro et al., 2013). Stott (2006) provides a review of various probability weighting functions and a summary of parameter values reported in the literature for each of the weighting functions. We refer the interested reader to this publication for descriptions of multiple weighting functions. Earlier research on probability weighting functions revealed considerable inter- and intra-individual variance of probability weighting parameters (Tversky and Kahneman, 1992; Gonzalez and Wu, 1999; Stott, 2006; Wu et al., 2009), but no former study addressed the question whether Bayesian inference in a risky/small world involves probability weighting through Bayesian modeling approaches.

The present study aimed at contributing to the literature by applying Bayesian statistics (Jaynes, 2003; MacKay, 2003; Robert, 2007) to model Bayesian inference. Bayesian methods have become increasingly accepted for data analysis in cognitive science (Edwards et al., 1963; Wagenmakers, 2007; Gallistel, 2009; Kruschke, 2010; Lee, 2011; Hoijtink, 2012). Specifically, hierarchical Bayesian modeling provides flexible and interpretable ways of analysing formal models of cognitive processes (Lee, 2011). Hierarchical Bayesian models are models in which parameters are sampled from distributions determined by other parameters (so-called hyper-parameters; Shiffrin et al., 2008). By making the individual parameters dependent on their group mean, a trade-off between fitting the group as a whole and fitting each individual separately is introduced (Shiffrin et al., 2008), allowing for improved predictive robustness of the model (Gelman et al., 2014a). Here, we applied hierarchical Bayesian modeling to Bayesian inference in order to examine whether human subjects (a) follow normative Bayesian specifications, and (b) are influenced by non-normative tendencies, such as base rate neglect or (inverse) S-shaped probability weighting. Notice that this is the first study that applied hierarchical Bayesian modeling to Bayesian inference in a risky/small world.

## Materials and methods

### Participants

Sixteen psychology students (*I* = 16) participated for course credit (15 female, 1 male). Age ranged from 19 to 50 years (*M* = 24.7; SD = 9.3). All participants indicated having normal or corrected-to-normal sight. The study was reviewed and approved by the Ethics Committee of TU Braunschweig (Department of Life Sciences). Informed consent was obtained from all subjects.

### Inference task

The inference task is a modification of tasks used in Phillips and Edwards (1966) and Grether (1980, 1992; see also Stern et al., 2010; Achtziger et al., 2014). Factorial combination of two levels of prior probabilities and two levels of the likelihoods yielded four experimental conditions which were administered to each participant. Their order was counterbalanced across participants under the following restriction: Prior probability was the slowly varying factor (one level of prior probabilities was repeated across two successive blocks of trials), whereas the likelihoods changed from block to block, yielding four different orders.

At the beginning of each condition, two types of urns (labeled *H*_1_ and *H*_2_ here, respectively) were presented on a computer screen. In uncertain likelihood conditions (*L*_*u*_), urn type *H*_1_ contained seven blue (*e* = 1) and three red (*e* = 0) balls [i.e., *P*(*e* = 1|*H*_1_) = 0.7, *P*(*e* = 0|*H*_1_) = 0.3], while urn type *H*_2_ contained three blue and seven red balls [i.e., *P*(*e* = 1|*H*_2_) = 0.3, *P*(*e* = 0|*H*_2_) = 0.7]. In certain likelihood conditions (*L*_*c*_), urn type *H*_1_ contained nine blue balls and one red ball [i.e., *P*(*e* = 1|*H*_1_) = 0.9, *P*(*e* = 0|*H*_1_) = 0.1], while urn type *H*_2_ contained one blue ball and nine red balls [i.e., *P*(*e* = 1|*H*_2_) = 0.1, *P*(*e* = 0|*H*_1_) = 0.9]. Prior probabilities were manipulated by presenting 10 urns, composed of different numbers of type *H*_1_ and type *H*_2_ urns. In uncertain prior probability conditions (*P*_*u*_), three type *H*_1_ and seven type *H*_2_ urns [i.e., *P*(*H*_1_) = 0.3, *P*(*H*_2_) = 0.7] were present. In the certain prior probability condition (*P*_*c*_), one type *H*_1_ urn and nine type *H*_2_ urns [i.e., *P*(*H*_1_) = 0.1, *P*(*H*_2_) = 0.9] were present. In the absence of a previous study that examined human probabilistic inference via hierarchical Bayesian modeling (see below), these quite un-balanced prior probabilities and likelihoods were chosen to minimize a potential role of pure “guessing.” It should be noted that the results may have been biased by the selection of these parameters. Colors were counterbalanced across participants, but we will ignore this in our description to avoid confusion.

Each experimental trial (Figure 1B) consisted of the following sequence of events: First, one urn was selected randomly, with the outcome of this selection remaining unknown to the participant. Subsequently, a random sample of four balls (*K* = 4) was drawn sequentially with replacement from that urn, shown one by one. Ignoring the order of drawn balls, this procedure generated five distinct possible situations (i.e., zero to four blue balls) in each condition. Ball stimuli were presented in the center of a computer screen (Eizo FlexScan T766 19″; Hakusan, Ishikawa, Japan) against gray background (size = one degree, duration = 100 ms, stimulus onset asynchrony = 2500 ms). Trial duration amounted to around 10 s. Participants were asked to indicate the color of each stimulus by pressing the left or right Ctrl key on a standard computer keyboard. Participants indicated which urn they considered more likely, given the number of blue balls drawn (zero to four), and based on the condition (prior probability and likelihood function; Data Sheet 1 in Supplementary Material). They indicated their inference by pressing the left or right Ctrl key for urn type *H*_1_ and urn type *H*_2_, respectively. Feedback was not provided in order to avoid confounding of probabilistic inference proper and evaluative processes, pending two-factor models of decision-making such as, for example, Prospect Theory (Kahneman and Tversky, 1979), although Grether and Plott (1979) have demonstrated that incentives may have little influence over performance in probabilistic inference tasks. Stimulus-response mapping was counterbalanced across participants. Neither feedback nor reward was provided during the experiment.

Each participant completed four practice trials under supervision of the experimenter, each with one urn type *H*_1_ and one urn type *H*_2_ exemplar, to become accustomed to the task. Successful completion of the practice trials demonstrated that participants understood the procedure and their task. There were *N* = 50 trials per condition, with short breaks between the conditions. We chose 50 trials per condition because the study was also designed to measure event-related potentials (Seer et al., 2016; Kopp et al., under review), and event-related potentials require large numbers of trials for averaging. Given five possible outcomes per condition, participants responded to each possible outcome an average of 10 times per condition. Thus, it should be mentioned that the responding had a quite repetitive character, and that participants may have at times simply recalled their responses from earlier trials in later trials. The experiment was run using Presentation® (Neurobehavioral Systems, Albany, CA).

### Bayesian inference

Binary inferences were requested (Figure 1). Given prior probabilities *P*(*H*_1_) = 1 − *P*(*H*_2_) for two hypothetical states *H*_1_ and *H*_2_, and a set of binary data *E* = {*e*_1_, …, *e*_*K*_} with *K* ∈ ℕ, we can compute the posterior probabilities as

(1) $$\begin{array}{r} P\left(H_{1}|E\right)=\frac{P\left(H_{1}\right)\cdot P\left(E|H_{1}\right)}{P\left(E\right)}, \end{array}$$

and

(2) $$\begin{array}{r} P\left(H_{2}|E\right)=\frac{P\left(H_{2}\right)\cdot P\left(E|H_{2}\right)}{P\left(E\right)}, \end{array}$$

where *P*(*H*_1_|*E*) = 1 − *P*(*H*_2_|*E*). Formulating these posterior probabilities in log-odds form (Jaynes, 2003), using the notation *Lo*(*P*(*H*_1_|*E*)) for the posterior log-odds favoring *H*_1_ we obtain,

(3) $$\begin{array}{r} Lo\left(P\left(H_{1}|E\right)\right)=\ln\;\frac{P\left(H_{1}\right)\cdot P\left(E|H_{1}\right)}{P\left(H_{2}\right)\cdot P\left(E|H_{2}\right)}\;. \end{array}$$

For each single binary datum *e*_*k*_, with *k* ∈ [1, …, *K*], we obtain,

(4) $$\begin{array}{r} Lo\left(P\left(H_{1}|E\right)\right)=\ln\;\frac{P\left(H_{1}\right)}{P\left(H_{2}\right)}\;+\overset{K}{\underset{k\;=\;1}{\sum }}\ln\;\frac{P\left(e_{k}|H_{1}\right)}{P\left(e_{k}|H_{2}\right)}\;. \end{array}$$

Hence, the Bayesian updating in log-odds form for a binary hypothesis equals adding the logarithm of the likelihood ratio ln *P*(*e*_*k*_|*H*_1_)∕*P*(*e*_*k*_|*H*_2_) to the logarithm of the prior odds ln *P*(*H*_1_)∕*P*(*H*_2_). We can therefore represent the accumulation of evidence by adding a new likelihood ratio for each new datum *e*_*k*_.

### Probability weighting

Zhang and Maloney (2012) proposed a linear probability weighting function in the log-odds space, capable of modeling probability weighting as in Prospect Theory (Kahneman and Tversky, 1979; Tversky and Kahneman, 1992). We chose this probability weighting function for its compatibility with the idea of Bayesian updating (see Section Bayesian Inference with Weighted Probabilities). Zhang and Maloney (2012) used the formula

(5) $$\begin{array}{r} \pi =\;logistic\left(\gamma \cdot Lo\left(p\right)+\left(1-\gamma \right)\cdot Lo\left(p_{0}\right)\right), \end{array}$$

with *p* being a probability and the two weighting-parameters γ ∈ [0, ∞) and *p*_0_ ∈ [0, 1], $\;logistic\left(x\right)=\frac{1}{1\;+\;e^{-x}}$ being the logistic function, π being the corresponding weighted probability, and *Lo*(*p*) given by

(6) $$\begin{array}{r} Lo\left(p\right)=\ln\;\frac{p}{1-p}\;. \end{array}$$

Note that, *logistic*(*Lo*(*p*)) = *p*.

The unknown parameters are the slope of the weighting function γ and the point *p*_0_, where the weighted probability equals the unweighted probability,

(7) $$\begin{array}{r} Lo\left(\pi \left(p_{0}\right)\right)=Lo\left(p_{0}\right). \end{array}$$

The slope parameter γ determines the shape of the weighting function. If γ = 1, the probabilities remain untransformed. If γ < 1, the weighting function has an inverse-S shape or a concave shape if *p*_0_ approaches one. If γ > 1, the weighting function shows an S-shape or a convex shape if *p*_0_ approaches one (Figure 2). Using a comparable log-odds approach, Zhang and Maloney (2012) successfully modeled choice and confidence data from a range of studies.

### Bayesian inference with weighted probabilities

Since Zhang and Maloney (2012) provide a linear weighting function in log-odds space, we can easily represent Bayesian inference with transformed log-odds. Applying Equation (5) to the log-odds evidence *Lo*(*P*(*H*_1_|*E*)) as defined in Equation (3) we obtain

(8) $$\begin{array}{r} Lo\left(\pi \left(H_{1}|E\right)\right)=\gamma \cdot \ln\;\frac{P\left(H_{1}\right)}{P\left(H_{2}\right)}\;+\gamma \cdot \ln\;\frac{P\left(E|H_{1}\right)}{P\left(E|H_{2}\right)}\;\\ +\;\left(1-\gamma \right)\cdot Lo\left(p_{0}\right). \end{array}$$

Therefore, we can also represent the weighted accumulation of evidence as the addition of

(9) $$\begin{array}{r} \gamma \cdot \ln\;\frac{P\left(E|H_{1}\right)}{P\left(E|H_{2}\right)}\;=\overset{K}{\underset{k\;=\;1}{\sum }}\gamma \cdot \ln\;\frac{P\left(e_{k}|H_{1}\right)}{P\left(e_{k}|H_{2}\right)}\;, \end{array}$$

for each single datum *e*_*k*_.

### Models

#### Unweighted Bayesian posterior probabilities

The simplest model incorporating Bayesian inference relies on unweighted posterior probabilities. This model is Bayes optimal, no other classifier of the urn state can do better on average. We use the posterior to predict the inference of *H*_1_, on any trial, using the sampling statement

(10) $$\begin{array}{r} y_{n}\;\sim \;bern\left(P\left(H_{1}|\;E\right)\right). \end{array}$$

Here, *y*_*n*_ represents the *n*-th inference (*y*_*n*_ ∈ {0, 1} with *n* ∈ {1, .., *N*}). A value of zero denotes the inference of the urn with higher prior probability and a value of one the inference of the urn with lower prior probability. *P*(*H*_1_|*E*) is the *n*-th posterior probability for *H*_1_, given a sample of evidence *E*, as defined in Equation (1). *bern*(*P*(*H*_1_|*E*)) denotes the Bernoulli distribution: *y*_*n*_ takes on value one with probability *P*(*H*_1_|*E*), and *y*_*n*_ equals zero with probability 1 − *P*(*H*_1_|*E*). There are five different possibilities to draw combinations of blue and red balls (disregarding sequential order); therefore there are five different values of *P*(*H*_1_|*E*) and *Lo*(*P*(*H*_1_|*E*)) (Equation 3) per experimental condition which are shown in Table 1.

**Table 1:** **Posterior probability for the rare urn *P*(*H*_1_|*E*) and *Lo*(*P*(*H*_1_|*E*)) for different conditions and different ball samples drawn**.

**Number of blue balls drawn**	**Condition**			
	***P*_*c*_*L*_*c*_**	***P*_*u*_*L*_*c*_**	***P*_*c*_*L*_*u*_**	***P*_*u*_*L*_*u*_**
***P*(*H*_1_|*E*)**				
Zero out of four	0.00002	0.00007	0.00373	0.01425
One out of four	0.00137	0.01526	0.02	0.07297
Two out of four	0.1	0.3	0.1	0.3
Three out of four	0.9	0.972	0.37692	0.7
Four out of four	0.99863	0.99964	0.76709	0.92703
***Lo*(*P*(*H*_1_|*E*))**				
Zero out of four	−10.99	−9.64	−5.59	−4.24
One out of four	−6.59	−5.24	−3.89	−2.54
Two out of four	−2.2	−0.85	−2.2	−0.85
Three out of four	2.2	3.55	−0.5	0.85
Four out of four	6.59	7.94	1.19	2.54

#### Base rate neglect

Base rate neglect leads to the assumption that π(*P*(*H*_1_)) = π(*P*(*H*_2_)) = 0.5, irrespective of the veridical prior probabilities *P*(*H*_1_) and *P*(*H*_2_). The model of Bayesian inference thus reduces to the likelihood of the data, i.e., to

(11) $$\begin{array}{r} y_{n}\;\sim \;bern\left(P\left(E|H_{1}\right)\right). \end{array}$$

#### Weighted Bayesian posterior probabilities

In the following, we describe three models of weighted Bayesian posterior probabilities (Equation 8).

##### Model without individual differences

This probability weighting model assumes the absence of inter-individual differences with regard to the weighting parameters γ and *p*_0_. The parameters γ und *p*_0_ were provided with weakly informative prior distributions—i.e., prior distributions restricting the posterior distribution very little—to ensure computational tractability (Gelman et al., 2014a).

**Figure 3:**
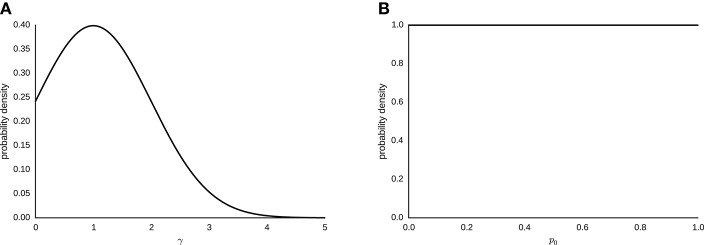
**(A)** Gaussian prior distribution for parameter γ ~ *N*(1, 1) for the interval [0, ∞). **(B)** Logit-normal prior distribution for parameter *p*_0_ ~ *beta*(1, 1).

The sampling statement for γ ~ *N*(1, 1) [0, ∞), with *N* being the normal distribution truncated at zero. Figure 3A depicts the corresponding probability density. The prior distribution of γ is a normal distribution centered on 1, to represent the weak prior assumption of no probability weighting taking place. We chose a flat prior *p*_0_ ~ *beta*(1, 1), where *beta* is the beta function. Figure 3B shows the corresponding probability density.

Thus, all urn inferences are generated by the underlying Bernoulli distribution

(12) $$\begin{array}{r} y_{n}\;\sim \;bern\left(logistic\left(\gamma \cdot Lo\left(p\right)+\left(1-\gamma \right)\cdot Lo\left(p_{0}\;\right)\right)\right), \end{array}$$

with *p* = *P*(*H*_1_|*E*), given a particular set of evidence *E*, *y*_*n*_ ∈ {0, 1} with *n* ∈ [1, …, *N*], γ ∈ [0, ∞) and *p*_0_ ∈ [0, 1]. *bern*(*p*_*b*_) denotes the Bernoulli distribution: *y*_*n*_ takes on value one—denoting an inference of the urn with lower prior probability—with probability *p*_*b*_, and *y*_*n*_ equals zero—an inference of the urn with higher prior probability—with probability 1 − *p*_*b*_.

**Figure 4:**
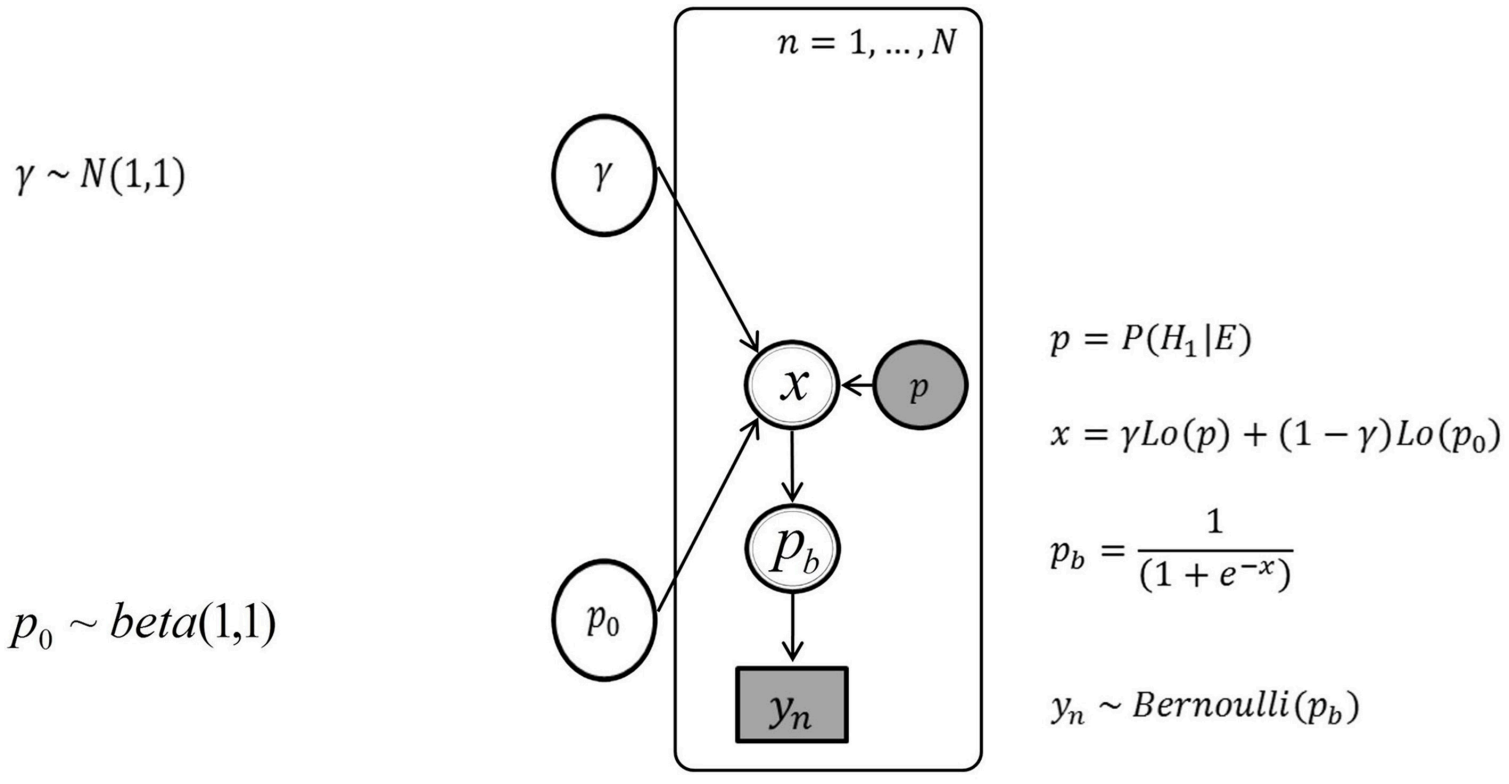
**The graph of the Bayesian network without inter-individual differences, with the urn inference *y*_*n*_, the posterior probability *p*, and the parameters *p*_0_ and γ**.

Figure 4 depicts the graphical model without individual differences. Nodes represent variables and arrows represent conditional probabilities. Discrete variables are given square nodes, continuous variables circular nodes and observed variables are shaded, while unobserved are not. Variables fully determined by their parents are given double borders, while stochastic variables are given single borders. Plate notation is used to ensure sparse representation, by grouping repeating variables in a subgraph enclosed by a rectangle, and indicating the number of repetitions. The plate corresponds to the *N* trials. An arrow between a variable outside of a plate to a variable inside indicates the dependence of each repeated variable on their parent outside the plate. The variables in the graphical model are defined using the subscript *n* to indicate definitions specific to the variables in each trial.

##### Model with unrestricted individual differences

This model considers inter-individual variability in parameters γ_*i*_ and *p*_0_*i*__. The sampling statements of the prior distributions of each individual's parameters γ_*i*_ and *p*_0_*i*__ are given by γ_*i*_ ~ *N*(1, 1) [0, ∞) and *p*_0_*i*__ ~ *beta*(1, 1).

The assumed data-generating process equals

(13) $$\begin{array}{r} {y_{n}}_{i}\;\sim \;bern\left(logistic\left(\gamma_{i}\cdot Lo\left(p\right)+\left(1-\gamma_{i}\right)\cdot Lo\left({p_{0}}_{i}\;\right)\right)\right), \end{array}$$

with *p* = *P*(*H*_1_|*E*), given the corresponding set of evidence *E*, *y*_*n*_*i*__ ∈ {0, 1}, γ_*i*_ ∈ [0, ∞), and *p*_0_*i*__ ∈ [0, 1] for each individual *i* and datum *n*. *bern*(*p*_*b*_) denotes the Bernoulli distribution.

**Figure 5:**
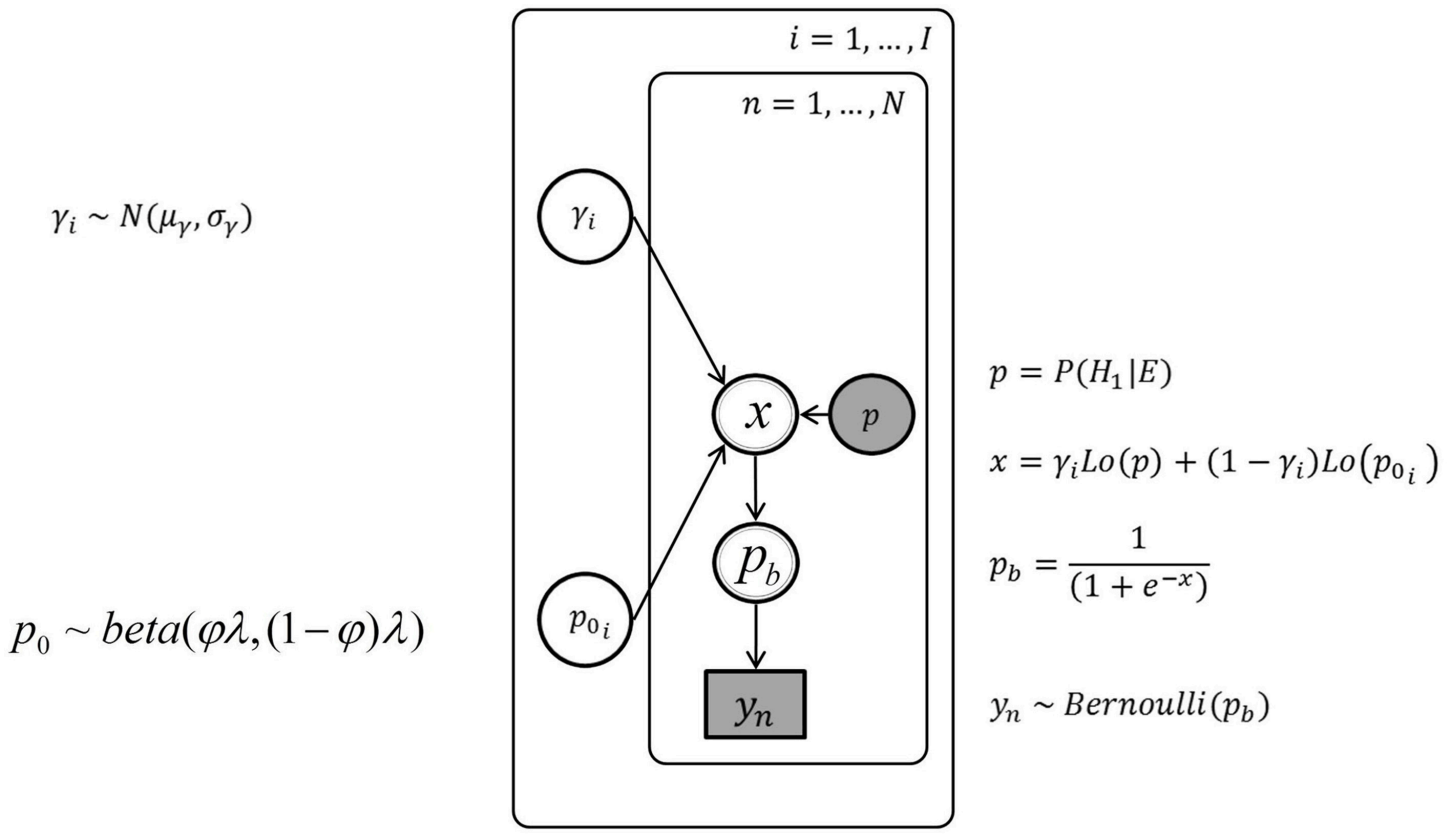
**The graph of the Bayesian network with unrestricted inter-individual differences, with the urn inference *y*_*n*_, the posterior probability *p*, and the individual parameters *p*_0*i*_ and γ_*i*_**.

Figure 5 shows the graphical model for the unrestricted inter-individual differences. The outer plate corresponds to the *I* = 16 individuals and the inner plate to the *N* = 50 trials. The variables in the graphical model are defined using the subscript *i* or *n* to indicate definitions for variables specific to each individual and trial, respectively.

##### Model with hierarchical individual differences

In this modeling attempt, we assume that the latent γ and *p*_0_ parameters for individual participants are generated by more abstract latent parameters (hyper-parameters) describing group distributions across individuals. This model considers individual differences by specifying distributions of parameters γ and *p*_0_, out of which individual γ_*i*_ and *p*_0*i*_ have to be sampled. For the parameter γ, these distributions were assumed to be normal distributions, characterized by mean μ_γ_ and standard deviation σ_γ_, with weakly informative prior distributions. The beta distribution of parameter *p*_0_ was specified by two hyper-parameters, one being a mean parameter φ = α∕(α + β) and one being a total count parameter λ = α + β, instead of the usual α and β, following Gelman et al. (2014b).

**Figure 6:**
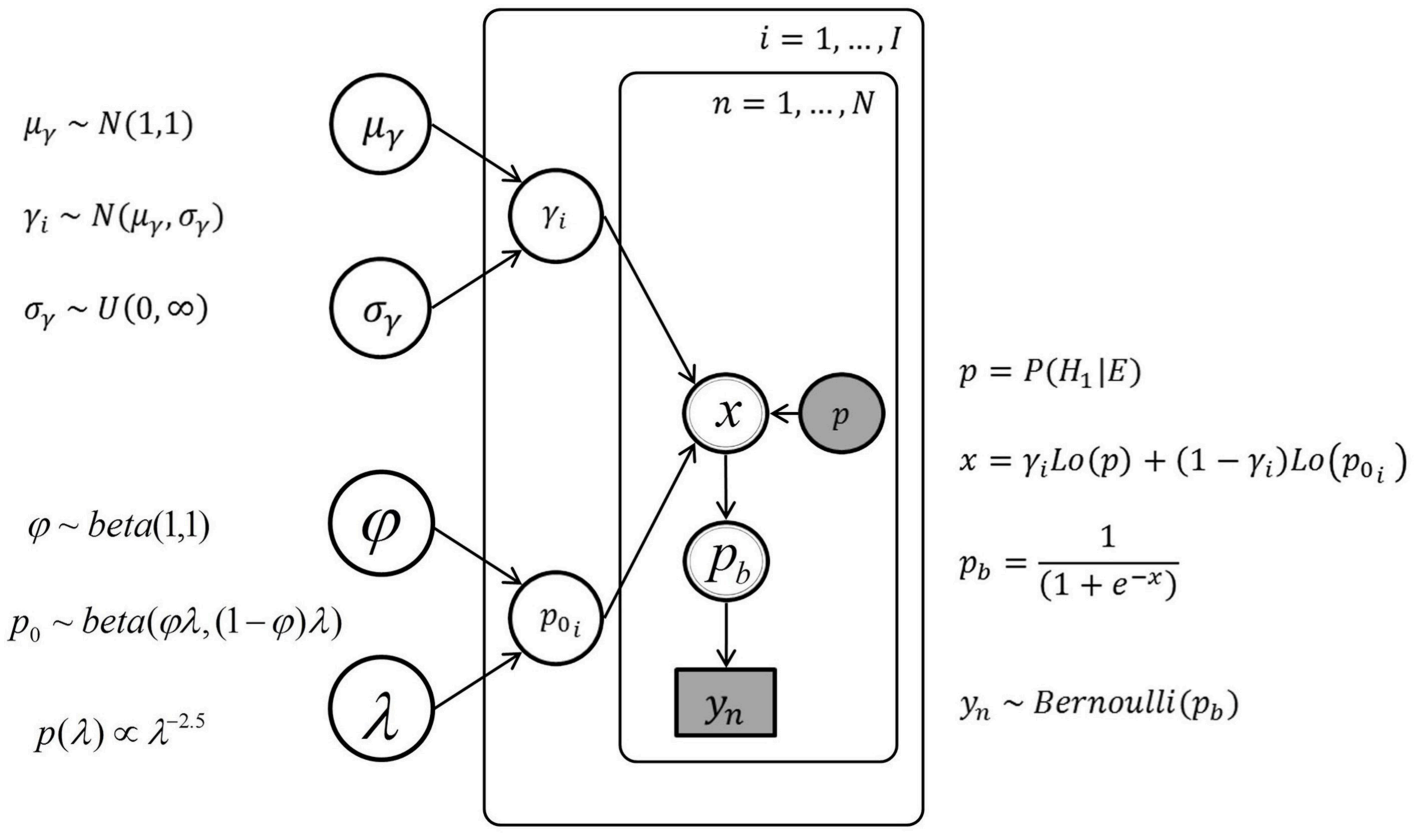
**The graph of the hierarchical Bayesian network, with the urn inference *y*_*n*_, the posterior probability *p*, the individual parameters *p*_0*i*_ and γ_*i*_, and the hyper-parameters μ_γ_, σ_γ_ and φ, λ**.

The sampling statement for the parameter γ_*i*_ for each individual *i* was γ_*i*_ ~ *N*(μ_γ_, σ_γ_)[0, ∞) with hyper-priors μ_γ_ ~ *N*(1, 1) and σ_γ_ ~ *Uniform*(0, ∞). The sampling statement for the parameter *p*_0_*i*__ for each individual *i* was *p*_0_*i*__ ~ *beta*(φλ, (1 − φ)λ) with φ ~ *beta*(1, 1) and λ having a pareto prior, *p*(λ)∝λ^−2.5^. The hierarchical model and the model with unrestricted individual differences share the same sampling state for the data (Equation 14), they differ only in the dependence (for the hierarchical model) or independence (for the model with unrestricted differences) of the γ_*i*_ and *p*_0*i*_ parameters. Figure 6 displays the graphical model for the hierarchical individual differences.

### Model evaluation and selection

The quality of the out-of-sample prediction of the models was evaluated by comparing their (a) *widely applicable information criterion* (WAIC; Watanabe, 2010) and (b) log-likelihood in a leave-one out cross-validation. For both measures, the log point-wise predictive density (lppd) was computed as,

(14) $$\begin{array}{r} lppd=\overset{N}{\underset{n\;=\;1}{\sum }}\ln\;\left(\frac{1}{S}\cdot \overset{S}{\underset{s\;=\;1}{\sum }}P\left(y_{n}|\theta^{s}\right)\right)\; \end{array}$$

Gelman et al. (2014b). This quantity measures the likelihood $P\left(y_{n}|\theta^{s}\right)$ of the datum *y*_*n*_, using *s* = 1, …, *S* sampling results from the posterior distribution of all model parameters, labeled θ^*s*^.

#### The widely applicable information criterion (WAIC)

The WAIC uses the log point-wise predictive density and subtracts a correction term, corresponding to the effective number of parameters (Gelman et al., 2014b), here given by

(15) $$\begin{array}{r} \overset{N}{\underset{n\;=\;1}{\sum }}V_{s\;=\;1}^{S}\left(\ln\;P\left(y_{n}|\;\theta^{s}\right)\right), \end{array}$$

with the likelihood $P\left(y_{n}|\theta^{s}\right)$ of the datum *y*_*n*_, using *s* = 1, …, *S* sampling results from the posterior distribution of all model parameters, labeled θ^*s*^, and the variance of the posterior,

!

Note that a_*s*_ is represented by $\ln\;P\left(y_{n}|\theta^{s}\right)\;$ in (17).

Subtracting (15) from (14) gives

(17) $$\begin{array}{r} \overset{N}{\underset{n\;=\;1}{\sum }}\ln\;\left(\frac{1}{S}\cdot \overset{S}{\underset{s\;=\;1}{\sum }}p\left(y_{n}|\theta^{s}\right)\right)\;-\overset{N}{\underset{n\;=\;1}{\sum }}V_{s\;=\;1}^{S}\left(\ln\;p\left(y_{n}|\;\theta^{s}\right)\right), \end{array}$$

which is the formula for calculating the WAIC (Gelman et al., 2014b). Multiplying the result from Equation (17) with −2 gives us a measure on the scale of other deviance and information measures like deviation information criterion (DIC; Spiegelhalter et al., 2002) and an information criterion (AIC; Akaike, 1973).

#### Bayesian leave-one-out cross-validation

In Bayesian leave-one-out cross-validation (Vehtari and Lampinen, 2002), each data point is predicted using the estimates of the posterior distributions obtained by fitting the model to all remaining data points. We fit the model with 15 individuals to predict the urn inferences of the 16-th person, since we are interested in predicting the urn inferences of additional individuals, rather than additional urn inferences of each individual. By doing this for every individual, we can calculate the sum of the individual log point-wise predictive densities. To make the interpretation of this score similar to other deviance measures, where smaller values denote a better fit, we use −2 · *lppd*. The model assuming unrestricted individual differences makes no prediction about the individual parameters γ and *p*_0_ for additional individuals. It is therefore impossible to cross-validate this model, and this model fails a basic test of generalizability because it does not make sensible predictions for the behavior of additional individuals. For the model without individual differences, using the posterior distributions of parameters γ and *p*_0_ is straightforward to compute the log posterior predictive density given in Equation (14). For the model with hierarchical individual differences, the unknown individual parameters γ and *p*_0_ were assumed to be sampled out of the hierarchical distributions characterized by the hyper-parameters μ_γ_, σ_γ_, φ, and λ.

The data were analyzed in Python 2.7.9 (Oliphant, 2007), and the models were fitted using the *Stan Hamiltonian-Monte-Carlo sampler* that provides approximate inference of the posterior distributions of the unknown parameters (Stan Development Team, 2013; Hoffman and Gelman, 2014). Each model in each condition was sampled with 10 chains and 20,000 iterations, with random initialization of the parameters and a warmup phase of 10,000 iterations (Data Sheet 2 in Supplementary Material).

## Results

### Data description

**Figure 7:**
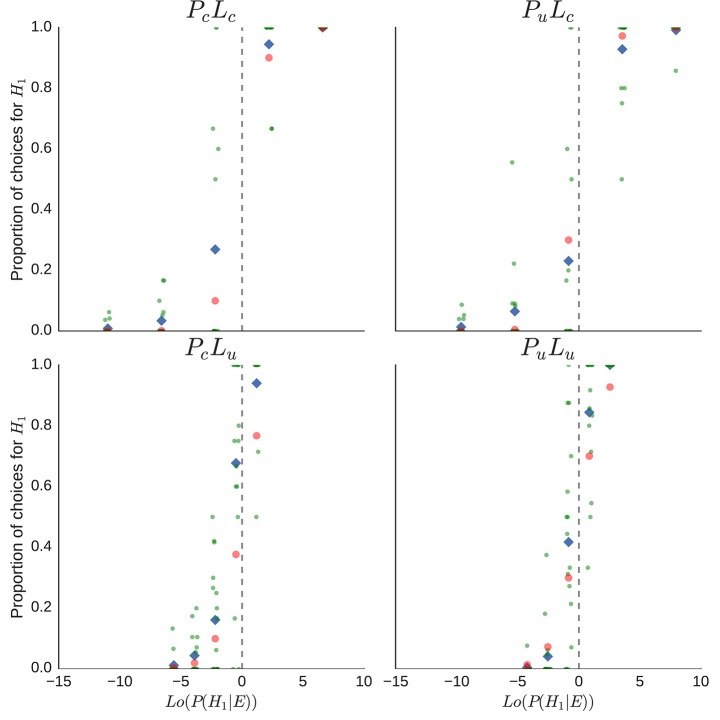
**Explorative data analysis for the individual proportions of *H*_1_-inferences per participant and sequence of drawn balls**. Each point represents the proportion of an individual per sequence, diamond shapes indicate the mean proportion of *H*_1_-inferences per sequence, and red dots indicate the appropriate proportion of *H*_1_-inferences, given the posterior probability *P*(*H*_1_|*E*). Overlapping proportions are “jittered” by adding small random noise on the x-axis.

In Figure 7, the proportions of *H*_1_-urn inferences are plotted separately for each condition (*P*_*c*_*L*_*c*_, *P*_*u*_*L*_*c*_, *P*_*c*_*L*_*u*_, *P*_*u*_*L*_*u*_) as a function of the log-odds posterior probabilities, *Lo*(*P*(*H*_1_|*E*)). Points represent the proportion from one participant; small random noise (±0.25) was added to the points to spread them out on the *x*-axis. Diamond symbols indicate mean proportions of inferences of *H*_1_ across participants, red dots indicate the proportion corresponding to the unweighted posterior probabilities. There is a considerable difference between the ideal proportion, indicated by the cross symbol, and the actual proportions of urn inferences of the participants, especially in the *P*_*c*_*L*_*u*_ condition, and there is also considerable variation between the individual proportions. Not represented are the different numbers of urn inferences which constituted the individual proportions.

### Parameter estimation

#### Unweighted Bayesian probabilities and base rate neglect

Predictions of the unweighted Bayesian model depend solely on posterior probabilities, *P*(*H*_1_|*E*), whereas predictions of the base rate neglect model depend solely on the likelihood of the data, *P*(*E*|*H*_1_). The predictions of these two models therefore do not depend on additional parameters. Further information about the quality of these predictions is given below under *Model Evaluation and Selection*.

#### Weighted Bayesian posterior probabilities

**Figure 8:**
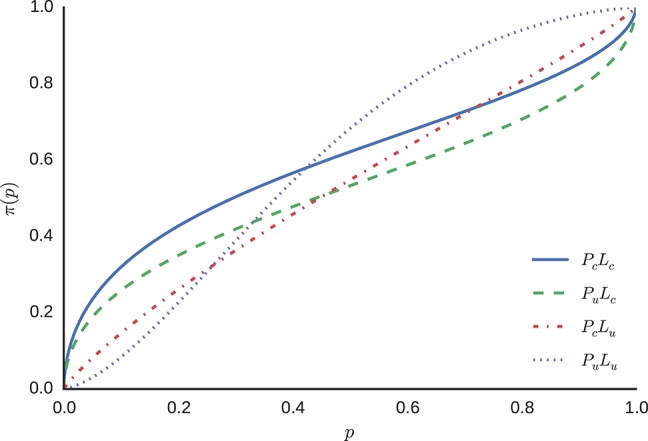
**Probability weighting functions using the posterior means for γ and *p*_0_**.

For the model without individual differences, sampling from the model described in Equation (12), yields the posterior distributions for parameters γ and *p*_0_ for each condition. The means of these posterior distributions were used as the estimates for the parameters in the weighting function described in Equation (5). The resulting functions vary across conditions, because the means of the posterior distributions for parameters γ and *p*_0_ differ between conditions. Figure 8 displays the resulting probability weighting functions.

The estimated weighted probability functions differ between conditions, indicating different regions of over- or under-representation of probabilities, with the exception of condition *P*_*c*_*L*_*u*_, where, according to the mean of the posterior distribution of the model, very little probability weighting takes place. Descriptive statistics of the fitted model without inter-individual differences are attached (see Appendix A in Supplementary Material).

**Figure 9:**
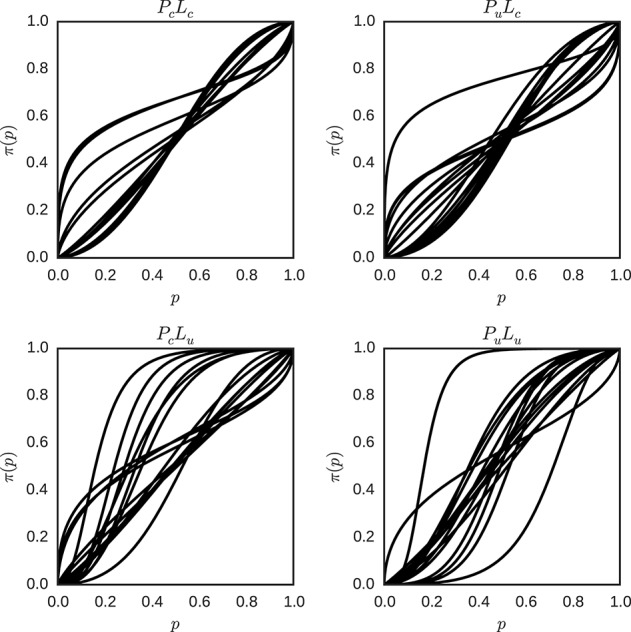
**Individual probability weighting functions using the posterior mean for γ and *p*_0_ with unrestricted inter-individual differences for the different conditions (*P*_*c*_*L*_*c*_, *P*_*u*_*L*_*c*_, *P*_*c*_*L*_*u*_, and *P*_*u*_*L*_*u*_)**. Each plot represents one condition, and each line represents the best estimate obtained for the weighted probability function for one individual in that condition.

For the model with unrestricted individual differences, sampling from the model described in Equation (13), yields the posterior distributions of the parameters specific for each individual in each condition. In each condition, the means of the posterior distributions of the individual parameters γ and *p*_0_ were used as the estimates of the parameters of the individual weighting functions described in Equation (5). Figure 9 shows the probability weighting functions for each individual and condition.

There is considerable variation in the shape of the probability weighting functions between individuals in one condition, and between conditions in general. Descriptive statistics of the fitted model with unrestricted inter-individual differences are attached (see Appendix B in Supplementary Material).

**Figure 10:**
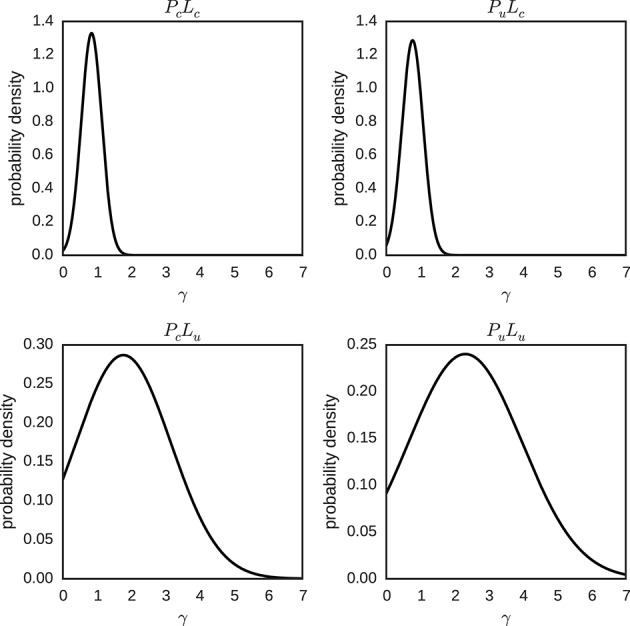
**Hierarchical distributions for parameter γ in different conditions (*P*_*c*_*L*_*c*_, *P*_*u*_*L*_*c*_, *P*_*c*_*L*_*u*_ and *P*_*u*_*L*_*u*_)**. The hierarchical distributions for γ ~ *N*(μ_γ_, σ_γ_) use the posterior means of the hyper-parameters μ_γ_ and σ_γ_.

For the model with hierarchical individual differences, the posterior distributions of each individual parameter γ and *p*_0_, and their hyper-parameters φ, λ, μ_γ_, and σ_γ_ for each condition were approximated by sampling. The means of the posterior distributions of μ_γ_ and σ_γ_, for each condition, were used to plot the expected hierarchical normal distribution of the individual parameters γ ~ *N*(μ_γ_, σ_γ_) in Figure 10. The posterior distribution of the γ parameter varies considerably between conditions. For conditions with a certain likelihood the posterior is centered around one, and is quite narrow. This is in contrast to the conditions with an uncertain likelihood, where the posterior is centered around higher values of γ and more spread out.

**Figure 11:**
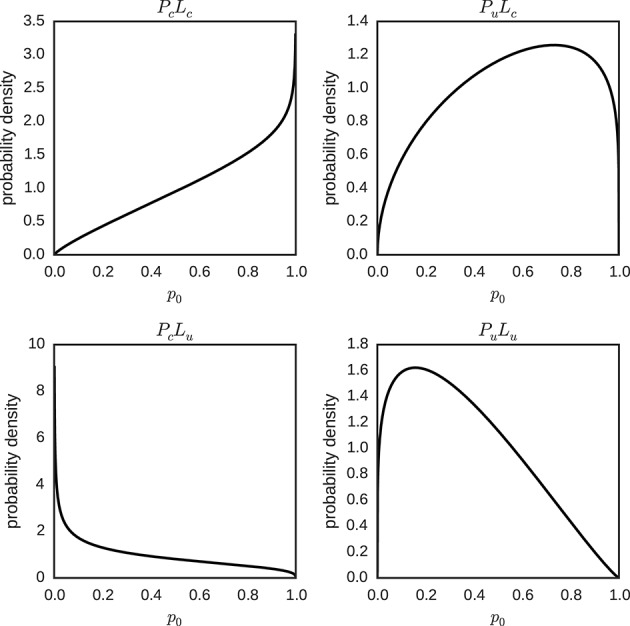
**Hierarchical distributions for parameter *p*_0_ in different conditions (*P*_*c*_*L*_*c*_, *P*_*u*_*L*_*c*_, *P*_*c*_*L*_*u*_ and *P*_*u*_*L*_*u*_)**. The hierarchical distributions *p*_0_ ~ *beta*(φλ, (1 − φ)λ) use the posterior means of the hyper-parameters φ and λ for the different conditions.

The means of the posterior distributions of φ and λ, for each condition, were used to plot the expected hierarchical beta distribution of the individual parameters *p*_0_ ~ *beta* (φλ, (1 − φ)λ) (Figure 11). We can distinguish two patterns in Figure 11: The posterior mode is (slightly) more extreme for certain than for uncertain prior conditions, and the posterior mode is closer to zero in conditions with an uncertain likelihood and closer to one in conditions with a certain likelihood.

**Figure 12:**
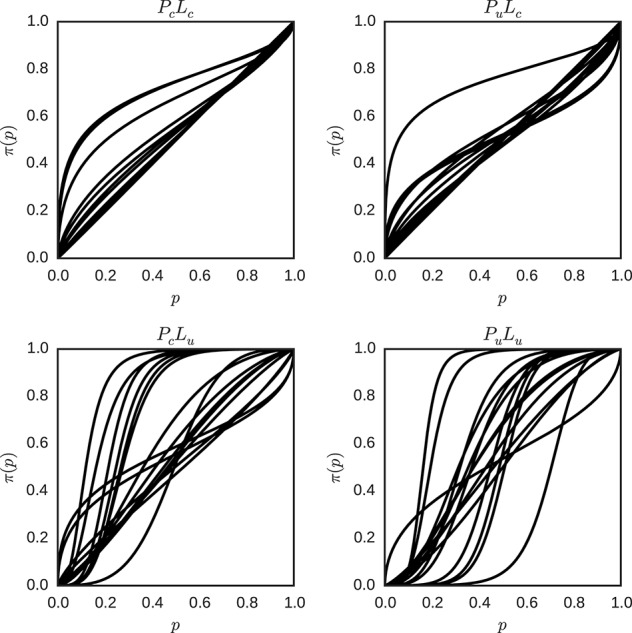
**Individual probability weighting functions using the posterior mean for γ and *p*_0_ in the hierarchical model for the different conditions (*P*_*c*_*L*_*c*_, *P*_*u*_*L*_*c*_, *P*_*c*_*L*_*u*_ and *P*_*u*_*L*_*u*_)**. Each plot represents one condition, and each line represents the best estimate obtained for the weighted probability function for one individual in that condition.

For the individual parameters γ and *p*_0_ for each individual and condition, the means of the posterior distributions were used as the parameters of the weighting function described in Equation (5), and the resulting functions were plotted in Figure 12.

There is still variation in the shape of the probability weighting functions between conditions, but the variation between individuals in one condition is reduced in contrast to Figure 9 for conditions with a more certain likelihood. Interestingly, in conditions with a more uncertain likelihood the variation between participants is either similar (*P*_*c*_*L*_*u*_) or larger (*P*_*u*_*L*_*u*_). Descriptive statistics of the fitted model with hierarchical inter-individual differences are attached (see Appendix C in Supplementary Material).

### Model evaluation and selection

#### Model comparison using the WAIC

Table 2 displays the WAICs for different models for each condition (*P*_*c*_*L*_*c*_, *P*_*u*_*L*_*c*_, *P*_*c*_*L*_*u*_, *P*_*u*_*L*_*u*_). Smaller values indicate a better out-of-sample prediction than larger values. A comparison between models reveals superior performance of weighted Bayesian models compared to unweighted models. In the latter category, both models have similar WAIC values on uncertain prior conditions *P*_*u*_, while the base rate neglect model performs better on the *P*_*c*_*L*_*c*_ condition, and the unweighted Bayesian model outperforms the base rate neglect model on the *P*_*c*_*L*_*u*_ condition. Among the weighted Bayesian models, those models with unrestricted individual differences and hierarchical individual differences perform better than the model without individual differences on all conditions. However, the hierarchical model outperforms the model with unrestricted inter-individual differences on *P*_*c*_*L*_*u*_ and *P*_*u*_*L*_*u*_ conditions, while both models perform similarly on the remaining conditions. In sum, the unweighted models yield larger WAIC values than the weighted models, with the hierarchical model providing the best overall performance.

**Table 2:** **WAIC deviance measures and standard errors for experimental conditions and models**.

**Model**	**Condition**			
	***P*_*c*_*L*_*c*_**	***P*_*u*_*L*_*c*_**	***P*_*c*_*L*_*u*_**	***P*_*u*_*L*_*u*_**
**Unweighted Bayesian**				
WAIC	157.90	207.70	257.38	293.58
SE	60.30	64.34	37.09	37.77
**Base rate neglect**				
WAIC	123.28	203.73	356.66	294.37
SE	32.33	54.62	36.00	28.67
**WEIGHTED BAYESIAN MODELS**				
**No differences**				
WAIC	93.90	139.93	228.48	263.24
SE	15.50	23.37	24.52	46.66
**Unrestricted differences**				
WAIC	71.59	118.03	162.20	187.89
SE	13.60	18.01	17.04	22.34
**Hierarchical differences**				
WAIC	73.08	121.60	152.77	163.08
SE	13.19	18.17	16.37	22.86

#### Model comparison using cross-validation

The results of the cross-validation, averaged over the 16 participants, are given in Table 3 for each condition (*P*_*c*_*L*_*c*_, *P*_*u*_*L*_*c*_, *P*_*c*_*L*_*u*_, *P*_*u*_*L*_*u*_). Further, cross-validation was not applicable to the model with unrestricted individual differences. The weighted Bayesian models predict new data better than the unweighted models. However, in the *P*_*u*_*L*_*u*_ condition, the model without inter-individual differences yields the worst average prediction of all models, while the model with hierarchical inter-individual differences still yields the best prediction. Among the weighted Bayesian models, the model with hierarchical individual differences outperforms the model without differences on conditions *P*_*u*_*L*_*c*_, *P*_*c*_*L*_*u*_, and *P*_*u*_*L*_*u*_, with especially large differences in the quality of prediction in uncertain likelihood conditions (*L*_*u*_), and similar performance of both models in the *P*_*c*_*L*_*c*_ condition.

**Table 3:** **Log posterior predictive density of leave-one-out cross-validation and standard errors for experimental conditions and models**.

**Model**	**Condition**			
	***P*_*c*_*L*_*c*_**	***P*_*u*_*L*_*c*_**	***P*_*c*_*L*_*u*_**	***P*_*u*_*L*_*u*_**
**Unweighted Bayesian**				
LOO-CV	157.92	207.68	257.44	293.60
SE	60.30	64.34	37.09	37.77
**Base rate neglect**				
LOO-CV	123.2	203.68	356.64	294.4
SE	32.33	54.61	36.00	28.66
**WEIGHTED BAYESIAN MODELS**				
**No differences**				
LOO-CV	89.28	133.12	215.36	248.64
SE	16.89	23.58	15.66	30.57
**Unrestricted differences**	n.a.	n.a.	n.a.	n.a.
** Hierarchical differences**				
LOO-CV	95.36	126.08	200.32	182.4
SE	22.74	20.66	27.02	15.18

### Effects of prior and likelihood manipulations

Since we observe differences in the probability weighting functions between conditions, we can test for the effect of the experimental prior and likelihood manipulations on the model parameters parameter γ and *p*_0_. We use the parameters of the model with full inter-individual differences, and estimate a linear mixed-effects model, with a random intercept per participant, categorical variables representing the manipulation of prior and likelihood and the interaction between these manipulations (see Appendix D in Supplementary Material for a more detailed description and the full model statistics). We estimate such a model separately for γ and *p*_0_ and find a significant effect for the uncertain likelihood conditions in γ (β = 0.35, *SE* = 0.17, *z* = 2.01, *p* = 0.036). Uncertain likelihood conditions increase the γ coefficient and change the probability weighting function to an S-shape (or a convex shape if *p*_0_ is close to one). For the *p*_0_ parameter, we find a significant effect also only for uncertain likelihood conditions (β = −0.15, *SE* = 0.05, *z* = −3.00, *p* = 0.003). In uncertain likelihood conditions the cutting point of the probability weighting function is lower than in certain likelihood conditions. Of course, this effect can also be seen in the posterior of the hierarchical model.

## Discussion

In this article, we explored various possibilities for modeling cognitive processes for probabilistic inference. To that end, probabilistic inference was observed in a small world (Savage, 1954), vested as a classic urn-ball paradigm (Phillips and Edwards, 1966; Grether, 1980, 1992; Stern et al., 2010; Achtziger et al., 2014) involving a factorial two (prior probabilities) by two (likelihoods) design. Probabilistic inference was modeled as originating from different variants of Bayesian inference. Five computational models of cognitive processes for probabilistic inference were compared by Bayesian model evaluation (Vehtari and Lampinen, 2002; Shiffrin et al., 2008). We found considerable task dependency such that more certain likelihoods were associated with mean group probability weighting parameters that satisfy γ < 0.6 (cf. Tables A.1, A.2), whereas less certain likelihoods were associated with mean group probability weighting parameters that satisfy γ < 0.9 (cf. Table A.3) or γ > 1 (cf. Table A.4). We are not aware of a psychological theory that could predict this strong task dependency of probability weighting.

The least complex models (i.e., parameter-free Bayesian posterior probabilities, parameter-free base rate neglect) provide inadequate models of probabilistic inference in terms of the robustness of the fit between models and data as well as in terms of their generalizability. The introduction of probability weighting functions (Kahneman and Tversky, 1979; Tversky and Kahneman, 1992; Prelec, 1998; Gonzalez and Wu, 1999; Luce, 2000; Zhang and Maloney, 2012; Cavagnaro et al., 2013) yielded more robust and generalizable fits between models and data than the two parameter-free models. Probability weighting models share the assumption that subjective probabilities deviate from true probabilities due to (inverted) S-shaped distortions of probabilities.

The least complex of these models conceptualized different slope, γ, and crossover point, *p*_0_, parameters for probability distortion across conditions, but not across individuals (model without individual differences). This model includes eight free parameters (four conditions by two free parameters, i.e., four γ and four *p*_0_ parameters).

There were two variants of individual difference models which envisaged different slope and crossover point parameters for probability distortion across conditions and individuals. The unrestricted individual differences model considered individual differences, by specifying individual γ_*i*_ and *p*_0_*i*__ parameters, which are fully determined by the data. This model includes four (conditions) by 32 free parameters (16 individual γ_*i*_ parameters and 16 individual *p*_0_*i*__ parameters, for a total of 128 free parameters).

In contrast, the hierarchical individual differences model assumed individual parameters themselves to be generated by more abstract latent parameters (hyper-parameters) describing group distributions across individuals. This model thus considered individual differences by specifying distributions of parameters γ and *p*_0_, out of which individual γ_*i*_ and *p*_0_*i*__ had to be sampled. This model includes four (conditions) by 32 free parameters (16 individual γ_*i*_ parameters and 16 individual *p*_0_*i*__ parameters), plus sixteen hyper-parameters (four conditions by four hyper-parameters, μ_γ_, σ_γ_, μ_*p*_0__, σ_*p*_0__, for a total of 144 parameters). Since the effective number of parameters in a hierarchical model depends on the variance of the group level parameters (Gelman et al., 2014b), the actual number of free parameters is < 144. We show that the hierarchical individual differences model outperformed the unrestricted individual differences model, which in turn outperformed the model without individual differences. This is the result of a process of model building, in which each increase in model complexity is justified by an increase in the model's fit and predictive power. To conclude, the assumption of large differences in probability distortions across tasks and individuals (i.e., in the values of the slope and crossover point parameters) seem critical for understanding Bayesian inference (see also Glöckner and Pachur, 2012; Zhang and Maloney, 2012). Further, it seems advantageous to consider individual differences as being sampled from weakly informative prior distributions of individual parameter values.

The hierarchical model of weighted Bayesian posterior probabilities is thus the preferable model of probabilistic inference, despite the non-negligible association between model performance and model complexity. However, parameter counts and estimations of model complexity should not be considered as equivalents. While the hierarchical model is more complex in terms of the number of parameters, it also restricts the individual parameters more than the model with unrestricted differences. Further, parameters, which are fully determined by the data (or by parts of the data, as in the unrestricted model), have a higher chance to overfit in comparison to those parameters that are imposed by some theoretical structure (e.g., the similarity of inter-individual differences, as in the hierarchical model). Similarly, to further prevent overfitting, the hierarchical model could be extended to explicitly model the different experimental conditions through an additional set of hyper-parameters. More concretely, an implementation in the framework of hierarchical Bayesian models would model the influence of subject, as well as experimental manipulation on the parameters of a probability weighting function. Further potential confounds, like task-order, and sequential effects in probability weighting might also be included. A final expansion of the hierarchical model could be its formulation as a Bayesian hierarchical mixture model (Bartlema et al., 2014), allowing groups of participants to have discrete, in addition to continuous, inter-individual differences.

Hierarchical Bayesian modeling offers specific advantages. Since knowledge about parameters propagates in hierarchical Bayesian models, the flow of probabilistic influence (Koller and Friedman, 2009) allows individual parameters to influence each other with regard to the certainty of their estimation. This renders the sharing of statistical strength (Gelman et al., 2014a) possible, with consequential improvements in predictive accuracy under high levels of uncertainty. This ubiquitous characteristic of hierarchical Bayesian modeling can also be recognized in our study, where the hierarchical model of weighted Bayesian posterior probabilities proved especially superior on those conditions that involved particularly high uncertainty about individual parameter values (i.e., the *L*_*u*_ conditions).

Our results are highly dependent on task conditions and individuals. However, the results from hierarchical Bayesian modeling converge with previous results in revealing that probability weighting parameters show considerable task dependency and individual differences (Tversky and Kahneman, 1992; Gonzalez and Wu, 1999; Stott, 2006; Wu et al., 2009). We have to admit that our work does not offer an a-priori explanation nor a post-hoc speculation as to why this should be the case. The hierarchical model is hence not a theory of cognitive processes for probabilistic inference, despite its hereby documented success in providing a reasonably good estimate of the observed data. Such a theory would encompass an answer to the question why subjective probabilities may deviate from the true probabilities in the ways that they apparently do, and specify the factors that affect this probability distortion. A full explanation of the phenomena just described would require not only that we account for the S-shaped form of the probability distortion, but also for the large differences in the values of the slope and crossover point parameters across tasks and individuals. Although such a theory still needs to be developed, we introduced hierarchical Bayesian modeling as a valuable method for developing a model, for testing it against data, for checking if there is systematic error in the predictions of the model, for increasing the complexity of the model as long as there are intolerable amounts of error, and for checking whether the more complex model provides a superior explanation for the observed data. Furthermore, hierarchical Bayesian modeling of cognitive processes for probabilistic inference may serve an instrumental role in neuroscientific studies of Bayesian inference (Kolossa et al., 2013, 2015).

## Conclusion

Our findings also bear on the question whether inductive inference (Anderson, 2015) can be described as being Bayesian or not (Gigerenzer and Murray, 1987; Koehler and Harvey, 2004). According to Clark (2013), the answer to this question is empirically underdetermined; thus, new methods for tackling the problem need to be developed and evaluated. Historically, two almost certainly irreconcilable intellectual camps evolved, although serious attempts exist to bridge the conflictive positions in the form of dual process models (Evans, 2008; Evans and Stanovich, 2013). The Bayesian camp claimed that there is sufficient evidence for postulating that Bayes' theorem can serve as a descriptive theory of human inductive inference (Chater et al., 2010; Tenenbaum et al., 2011; Griffiths et al., 2012). However, the non-Bayesian camp insisted that the evidence showed that humans critically deviate from prescriptive Bayesian solutions (e.g., base rate neglect; Kahneman and Tversky, 1973; Bar-Hillel, 1980). In fact, some researchers believe that humans should be better described as *homo heuristicus* rather than as rational probabilists (Kahneman et al., 1982; Gigerenzer and Brighton, 2009). Our data offer another possibility: Humans might be able to integrate information for inductive inference according to Bayesian prescriptions (cf. Equations 1–4), yet in terms of distorted probabilities (cf. Equations 5–9), as originally conjectured by Edwards (1954) and by Prospect Theory (Kahneman and Tversky, 1979; Tversky and Kahneman, 1992).

## Author contributions

BK developed the study design and collected the data. MB performed the data analysis under the supervision of BK. MB and BK drafted the manuscript, and CS and FL provided critical revisions. All authors approved the final version of the manuscript for submission.

## Funding

The research reported here was supported by a grant to BK from the Petermax-Müller-Stiftung, Hannover, Germany. In addition, FL received funding from the German National Academic Foundation.

### Conflict of interest statement

The authors declare that the research was conducted in the absence of any commercial or financial relationships that could be construed as a potential conflict of interest.
