# Supplementary material for: Probabilistic Inference: Task Dependency and Individual Differences of Probability Weighting Revealed by Hierarchical Bayesian Modeling
Source: Front Psychol. 2016 May 27;7:755. doi: 10.3389/fpsyg.2016.00755 (PMC4882416; doi:10.3389/fpsyg.2016.00755)
Supplement: Supplementary file 1 [file DataSheet1.docx]

**Supporting Online Material**

**Appendix A**

Sampling output of the posterior distributions of the parameters $\gamma$ and $p_{0}$ of the model without inter-individual differences. $\hat{R}$ is a measure for convergence of different sampling chains (Gelman & Rubin, 1992), at convergence $\hat{R}=1$.

Table A.1

*Posterior distributions of parameters* $\gamma$ *and* $p_{0}$ *for condition* $P_{c}L_{c}$***.***

|  | *M* | *SE* | *SD* | 2.5% | 25% | 50% | 75% | 97.5% | $\hat{R}$ |
| --- | --- | --- | --- | --- | --- | --- | --- | --- | --- |
| $\gamma$ | 0.57 | 0.00 | 0.05 | 0.48 | 0.54 | 0.57 | 0.6 | 0.67 | 1.00 |
| $p_{0}$ | 0.76 | 0.00 | 0.11 | 0.52 | 0.69 | 0.77 | 0.85 | 0.95 | 1.00 |

Table A.2

*Posterior distributions of parameters* $\gamma$ *and* $p_{0}$ *for condition* $P_{u}L_{c}$*.*

|  | *M* | *SE* | *SD* | 2.5% | 25% | 50% | 75% | 97.5% | $\hat{R}$ |
| --- | --- | --- | --- | --- | --- | --- | --- | --- | --- |
| $\gamma$ | 0.54 | 0.00 | 0.04 | 0.47 | 0.52 | 0.54 | 0.57 | 0.63 | 1.00 |
| $p_{0}$ | 0.57 | 0.00 | 0.10 | 0.39 | 0.51 | 0.57 | 0.64 | 0.76 | 1.00 |

Table A.3

*Posterior distributions of parameters* $\gamma$ *and* $p_{0}$ *for condition* $P_{c}L_{u}$*.*

|  | *M* | *SE* | *SD* | 2.5% | 25% | 50% | 75% | 97.5% | $\hat{R}$ |
| --- | --- | --- | --- | --- | --- | --- | --- | --- | --- |
| $\gamma$ | 0.89 | 0.00 | 0.12 | 0.77 | 0.83 | 0.86 | 0.89 | 1.26 | 1.60 |
| $p_{0}$ | 0.85 | 0.00 | 0.29 | 0.00 | 0.89 | 0.95 | 0.98 | 1.00 | 1.90 |

Table A.4

*Posterior distributions of parameters* $\gamma$ *and* $p_{0}$ *for condition* $P_{u}L_{u}$*.*

|  | *M* | *SE* | *SD* | 2.5% | 25% | 50% | 75% | 97.5% | $\hat{R}$ |
| --- | --- | --- | --- | --- | --- | --- | --- | --- | --- |
| $\gamma$ | 1.44 | 0 | 0.1 | 1.25 | 1.37 | 1.44 | 1.51 | 1.65 | 1.00 |
| $p_{0}$ | 0.15 | 0 | 0.04 | 0.06 | 0.12 | 0.15 | 0.18 | 0.23 | 1.00 |

**Appendix B**

Sampling output of the posterior distributions of the parameters $\gamma$ and $p_{0}$ of each individual with unrestricted inter-individual differences. $\hat{R}$ is a measure for convergence of different sampling chains (Gelman & Rubin, 1992), at convergence $\hat{R}=1$.

Table B.1

*Posterior distributions of each individual parameter* $\gamma$ *and* $p_{0}$ *for condition* $P_{c}L_{c}$*.*

|  | *M* | *SE* | *SD* | 2.5% | 25% | 50% | 75% | 97.5% | $\hat{R}$ |
| --- | --- | --- | --- | --- | --- | --- | --- | --- | --- |
| $\gamma_{1}$ | 1.12 | 0.00 | 0.58 | 0.42 | 0.71 | 0.96 | 1.40 | 2.63 | 1.00 |
| $\gamma_{2}$ | 0.64 | 0.00 | 0.21 | 0.33 | 0.49 | 0.61 | 0.75 | 1.16 | 1.00 |
| $\gamma_{3}$ | 1.61 | 0.00 | 0.68 | 0.60 | 1.08 | 1.51 | 2.03 | 3.17 | 1.00 |
| $\gamma_{4}$ | 0.43 | 0.00 | 0.11 | 0.21 | 0.35 | 0.43 | 0.50 | 0.65 | 1.00 |
| $\gamma_{5}$ | 1.18 | 0.00 | 0.57 | 0.44 | 0.76 | 1.04 | 1.46 | 2.61 | 1.00 |
| $\gamma_{6}$ | 0.37 | 0.00 | 0.09 | 0.20 | 0.31 | 0.37 | 0.43 | 0.55 | 1.00 |
| $\gamma_{7}$ | 1.24 | 0.00 | 0.70 | 0.41 | 0.71 | 1.03 | 1.61 | 3.01 | 1.00 |
| $\gamma_{8}$ | 0.69 | 0.00 | 0.23 | 0.33 | 0.53 | 0.66 | 0.81 | 1.23 | 1.00 |
| $\gamma_{9}$ | 1.54 | 0.00 | 0.70 | 0.52 | 0.99 | 1.43 | 1.97 | 3.15 | 1.00 |
| $\gamma_{10}$ | 1.69 | 0.00 | 0.67 | 0.67 | 1.19 | 1.60 | 2.10 | 3.22 | 1.00 |
| $\gamma_{11}$ | 1.62 | 0.00 | 0.69 | 0.57 | 1.10 | 1.53 | 2.06 | 3.19 | 1.00 |
| $\gamma_{12}$ | 1.71 | 0.00 | 0.67 | 0.67 | 1.20 | 1.62 | 2.12 | 3.24 | 1.00 |
| $\gamma_{13}$ | 0.40 | 0.00 | 0.09 | 0.23 | 0.33 | 0.39 | 0.46 | 0.58 | 1.00 |
| $\gamma_{14}$ | 1.56 | 0.00 | 0.69 | 0.53 | 1.03 | 1.46 | 1.99 | 3.14 | 1.00 |
| $\gamma_{15}$ | 1.66 | 0.00 | 0.67 | 0.64 | 1.15 | 1.57 | 2.08 | 3.20 | 1.00 |
| $\gamma_{16}$ | 1.23 | 0.00 | 0.58 | 0.47 | 0.81 | 1.10 | 1.54 | 2.67 | 1.00 |
| ${p_{0}}_{1}$ | 0.43 | 0.00 | 0.30 | 0.01 | 0.16 | 0.41 | 0.68 | 0.96 | 1.00 |
| ${p_{0}}_{2}$ | 0.58 | 0.00 | 0.25 | 0.08 | 0.39 | 0.60 | 0.79 | 0.97 | 1.00 |
| ${p_{0}}_{3}$ | 0.49 | 0.00 | 0.28 | 0.03 | 0.25 | 0.48 | 0.72 | 0.96 | 1.00 |
| ${p_{0}}_{4}$ | 0.65 | 0.00 | 0.22 | 0.20 | 0.48 | 0.67 | 0.83 | 0.98 | 1.00 |
| ${p_{0}}_{5}$ | 0.56 | 0.00 | 0.30 | 0.04 | 0.30 | 0.58 | 0.84 | 0.98 | 1.00 |
| ${p_{0}}_{6}$ | 0.76 | 0.00 | 0.17 | 0.38 | 0.65 | 0.79 | 0.89 | 0.98 | 1.00 |
| ${p_{0}}_{7}$ | 0.42 | 0.00 | 0.32 | 0.00 | 0.10 | 0.40 | 0.71 | 0.97 | 1.00 |
| ${p_{0}}_{8}$ | 0.52 | 0.00 | 0.26 | 0.06 | 0.30 | 0.52 | 0.73 | 0.96 | 1.00 |
| ${p_{0}}_{9}$ | 0.49 | 0.00 | 0.29 | 0.03 | 0.24 | 0.49 | 0.74 | 0.97 | 1.00 |
| ${p_{0}}_{10}$ | 0.50 | 0.00 | 0.27 | 0.04 | 0.26 | 0.49 | 0.72 | 0.96 | 1.00 |
| ${p_{0}}_{11}$ | 0.51 | 0.00 | 0.28 | 0.04 | 0.27 | 0.51 | 0.75 | 0.97 | 1.00 |
| ${p_{0}}_{12}$ | 0.49 | 0.00 | 0.27 | 0.04 | 0.26 | 0.49 | 0.72 | 0.95 | 1.00 |
| ${p_{0}}_{13}$ | 0.77 | 0.00 | 0.16 | 0.41 | 0.67 | 0.80 | 0.90 | 0.99 | 1.00 |
| ${p_{0}}_{14}$ | 0.50 | 0.00 | 0.28 | 0.03 | 0.26 | 0.50 | 0.74 | 0.97 | 1.00 |
| ${p_{0}}_{15}$ | 0.51 | 0.00 | 0.27 | 0.04 | 0.28 | 0.51 | 0.74 | 0.96 | 1.00 |
| ${p_{0}}_{16}$ | 0.57 | 0.00 | 0.30 | 0.04 | 0.30 | 0.60 | 0.85 | 0.98 | 1.00 |

Table B.2

*Posterior distributions of each individual parameter* $\gamma$ *and* $p_{0}$ *for condition* $P_{u}L_{c}$*.*

|  | *M* | *SE* | *SD* | 2.5% | 25% | 50% | 75% | 97.5% | $\hat{R}$ |
| --- | --- | --- | --- | --- | --- | --- | --- | --- | --- |
| $\gamma_{1}$ | 1.62 | 0.00 | 0.67 | 0.61 | 1.11 | 1.52 | 2.03 | 3.15 | 1.00 |
| $\gamma_{2}$ | 1.46 | 0.00 | 0.57 | 0.62 | 1.04 | 1.36 | 1.78 | 2.81 | 1.00 |
| $\gamma_{3}$ | 1.08 | 0.00 | 0.62 | 0.42 | 0.64 | 0.86 | 1.35 | 2.73 | 1.00 |
| $\gamma_{4}$ | 0.39 | 0.00 | 0.10 | 0.23 | 0.32 | 0.38 | 0.45 | 0.61 | 1.00 |
| $\gamma_{5}$ | 0.50 | 0.00 | 0.13 | 0.29 | 0.41 | 0.49 | 0.58 | 0.78 | 1.00 |
| $\gamma_{6}$ | 0.39 | 0.00 | 0.10 | 0.22 | 0.32 | 0.38 | 0.45 | 0.61 | 1.00 |
| $\gamma_{7}$ | 1.72 | 0.00 | 0.67 | 0.70 | 1.22 | 1.63 | 2.14 | 3.24 | 1.00 |
| $\gamma_{8}$ | 0.75 | 0.00 | 0.25 | 0.37 | 0.57 | 0.71 | 0.89 | 1.35 | 1.00 |
| $\gamma_{9}$ | 0.39 | 0.00 | 0.08 | 0.24 | 0.33 | 0.39 | 0.44 | 0.56 | 1.00 |
| $\gamma_{10}$ | 1.70 | 0.00 | 0.66 | 0.72 | 1.21 | 1.61 | 2.11 | 3.21 | 1.00 |
| $\gamma_{11}$ | 0.61 | 0.00 | 0.17 | 0.34 | 0.49 | 0.59 | 0.72 | 1.01 | 1.00 |
| $\gamma_{12}$ | 1.53 | 0.00 | 0.57 | 0.68 | 1.12 | 1.44 | 1.86 | 2.89 | 1.00 |
| $\gamma_{13}$ | 0.79 | 0.00 | 0.36 | 0.41 | 0.59 | 0.70 | 0.87 | 1.84 | 1.00 |
| $\gamma_{14}$ | 1.63 | 0.00 | 0.67 | 0.64 | 1.12 | 1.53 | 2.04 | 3.17 | 1.00 |
| $\gamma_{15}$ | 1.56 | 0.00 | 0.69 | 0.59 | 1.02 | 1.44 | 1.98 | 3.15 | 1.00 |
| $\gamma_{16}$ | 0.83 | 0.00 | 0.48 | 0.40 | 0.57 | 0.69 | 0.86 | 2.39 | 1.00 |
| ${p_{0}}_{1}$ | 0.54 | 0.00 | 0.27 | 0.05 | 0.32 | 0.56 | 0.78 | 0.97 | 1.00 |
| ${p_{0}}_{2}$ | 0.49 | 0.00 | 0.25 | 0.05 | 0.29 | 0.48 | 0.69 | 0.95 | 1.00 |
| ${p_{0}}_{3}$ | 0.49 | 0.00 | 0.35 | 0.01 | 0.17 | 0.44 | 0.85 | 1.00 | 1.00 |
| ${p_{0}}_{4}$ | 0.49 | 0.00 | 0.18 | 0.16 | 0.36 | 0.49 | 0.62 | 0.85 | 1.00 |
| ${p_{0}}_{5}$ | 0.57 | 0.00 | 0.22 | 0.16 | 0.41 | 0.57 | 0.74 | 0.94 | 1.00 |
| ${p_{0}}_{6}$ | 0.50 | 0.00 | 0.18 | 0.18 | 0.37 | 0.49 | 0.62 | 0.85 | 1.00 |
| ${p_{0}}_{7}$ | 0.58 | 0.00 | 0.26 | 0.07 | 0.38 | 0.60 | 0.80 | 0.97 | 1.00 |
| ${p_{0}}_{8}$ | 0.48 | 0.00 | 0.26 | 0.04 | 0.25 | 0.46 | 0.69 | 0.95 | 1.00 |
| ${p_{0}}_{9}$ | 0.87 | 0.00 | 0.10 | 0.63 | 0.82 | 0.89 | 0.95 | 0.99 | 1.00 |
| ${p_{0}}_{10}$ | 0.55 | 0.00 | 0.27 | 0.06 | 0.33 | 0.56 | 0.78 | 0.97 | 1.00 |
| ${p_{0}}_{11}$ | 0.52 | 0.00 | 0.23 | 0.08 | 0.34 | 0.52 | 0.70 | 0.94 | 1.00 |
| ${p_{0}}_{12}$ | 0.52 | 0.00 | 0.24 | 0.07 | 0.33 | 0.51 | 0.71 | 0.95 | 1.00 |
| ${p_{0}}_{13}$ | 0.61 | 0.00 | 0.29 | 0.01 | 0.40 | 0.67 | 0.85 | 0.99 | 1.00 |
| ${p_{0}}_{14}$ | 0.49 | 0.00 | 0.28 | 0.03 | 0.25 | 0.49 | 0.74 | 0.97 | 1.00 |
| ${p_{0}}_{15}$ | 0.37 | 0.00 | 0.28 | 0.01 | 0.12 | 0.31 | 0.58 | 0.94 | 1.00 |
| ${p_{0}}_{16}$ | 0.40 | 0.00 | 0.30 | 0.02 | 0.15 | 0.33 | 0.61 | 1.00 | 1.00 |

Table B.3

*Posterior distributions of each individual parameter* $\gamma$ *and* $p_{0}$ *for condition* $P_{c}L_{u}$*.*

|  | *M* | *SE* | *SD* | 2.5% | 25% | 50% | 75% | 97.5% | $\hat{R}$ |
| --- | --- | --- | --- | --- | --- | --- | --- | --- | --- |
| $\gamma_{1}$ | 2.55 | 0.00 | 0.68 | 1.33 | 2.07 | 2.52 | 2.99 | 3.97 | 1.00 |
| $\gamma_{2}$ | 1.12 | 0.00 | 0.33 | 0.53 | 0.90 | 1.09 | 1.30 | 1.86 | 1.00 |
| $\gamma_{3}$ | 1.90 | 0.00 | 0.69 | 0.70 | 1.45 | 1.89 | 2.34 | 3.32 | 1.00 |
| $\gamma_{4}$ | 1.22 | 0.00 | 0.33 | 0.70 | 0.99 | 1.18 | 1.41 | 2.00 | 1.00 |
| $\gamma_{5}$ | 0.94 | 0.00 | 0.34 | 0.25 | 0.71 | 0.95 | 1.16 | 1.62 | 1.00 |
| $\gamma_{6}$ | 0.59 | 0.00 | 0.18 | 0.31 | 0.50 | 0.59 | 0.67 | 0.83 | 1.33 |
| $\gamma_{7}$ | 2.71 | 0.00 | 0.67 | 1.51 | 2.24 | 2.67 | 3.14 | 4.12 | 1.00 |
| $\gamma_{8}$ | 1.97 | 0.00 | 0.63 | 0.98 | 1.51 | 1.89 | 2.35 | 3.40 | 1.00 |
| $\gamma_{9}$ | 0.51 | 0.00 | 0.14 | 0.20 | 0.41 | 0.51 | 0.61 | 0.76 | 1.00 |
| $\gamma_{10}$ | 0.87 | 0.00 | 0.37 | 0.47 | 0.65 | 0.74 | 0.88 | 1.86 | 1.00 |
| $\gamma_{11}$ | 2.16 | 0.00 | 0.71 | 0.96 | 1.63 | 2.11 | 2.62 | 3.67 | 1.00 |
| $\gamma_{12}$ | 2.29 | 0.00 | 0.61 | 0.84 | 1.90 | 2.25 | 2.66 | 3.58 | 1.00 |
| $\gamma_{13}$ | 0.48 | 0.00 | 0.13 | 0.21 | 0.40 | 0.49 | 0.58 | 0.72 | 1.00 |
| $\gamma_{14}$ | 1.35 | 0.00 | 0.46 | 0.69 | 1.01 | 1.27 | 1.62 | 2.43 | 1.00 |
| $\gamma_{15}$ | 2.40 | 0.00 | 0.72 | 1.10 | 1.88 | 2.37 | 2.88 | 3.90 | 1.00 |
| $\gamma_{16}$ | 1.19 | 0.00 | 0.37 | 0.56 | 0.94 | 1.14 | 1.39 | 2.06 | 1.00 |
| ${p_{0}}_{1}$ | 0.18 | 0.00 | 0.09 | 0.05 | 0.12 | 0.16 | 0.22 | 0.40 | 1.00 |
| ${p_{0}}_{2}$ | 0.44 | 0.00 | 0.26 | 0.03 | 0.22 | 0.41 | 0.64 | 0.95 | 1.00 |
| ${p_{0}}_{3}$ | 0.15 | 0.00 | 0.23 | 0.01 | 0.04 | 0.06 | 0.10 | 0.89 | 1.00 |
| ${p_{0}}_{4}$ | 0.46 | 0.00 | 0.25 | 0.05 | 0.26 | 0.44 | 0.65 | 0.94 | 1.00 |
| ${p_{0}}_{5}$ | 0.39 | 0.00 | 0.29 | 0.02 | 0.13 | 0.34 | 0.62 | 0.95 | 1.00 |
| ${p_{0}}_{6}$ | 0.75 | 0.00 | 0.20 | 0.29 | 0.64 | 0.80 | 0.91 | 0.99 | 1.34 |
| ${p_{0}}_{7}$ | 0.14 | 0.00 | 0.07 | 0.04 | 0.10 | 0.13 | 0.17 | 0.28 | 1.00 |
| ${p_{0}}_{8}$ | 0.56 | 0.00 | 0.22 | 0.12 | 0.40 | 0.57 | 0.74 | 0.94 | 1.00 |
| ${p_{0}}_{9}$ | 0.66 | 0.00 | 0.19 | 0.29 | 0.52 | 0.66 | 0.81 | 0.97 | 1.00 |
| ${p_{0}}_{10}$ | 0.62 | 0.00 | 0.33 | 0.00 | 0.43 | 0.73 | 0.89 | 0.99 | 1.00 |
| ${p_{0}}_{11}$ | 0.25 | 0.00 | 0.17 | 0.05 | 0.13 | 0.20 | 0.31 | 0.75 | 1.00 |
| ${p_{0}}_{12}$ | 0.05 | 0.00 | 0.13 | 0.00 | 0.02 | 0.03 | 0.04 | 0.46 | 1.00 |
| ${p_{0}}_{13}$ | 0.70 | 0.00 | 0.18 | 0.33 | 0.57 | 0.72 | 0.85 | 0.98 | 1.00 |
| ${p_{0}}_{14}$ | 0.33 | 0.00 | 0.26 | 0.02 | 0.11 | 0.24 | 0.51 | 0.92 | 1.00 |
| ${p_{0}}_{15}$ | 0.23 | 0.00 | 0.13 | 0.07 | 0.14 | 0.19 | 0.27 | 0.61 | 1.00 |
| ${p_{0}}_{16}$ | 0.40 | 0.00 | 0.25 | 0.04 | 0.19 | 0.35 | 0.58 | 0.93 | 1.00 |

Table B.4

*Posterior distributions of each individual parameter* $\gamma$ *and* $p_{0}$ *for condition* $P_{u}L_{u}$*.*

|  | *M* | *SE* | *SD* | 2.5% | 25% | 50% | 75% | 97.5% | $\hat{R}$ |
| --- | --- | --- | --- | --- | --- | --- | --- | --- | --- |
| $\gamma_{1}$ | 2.72 | 0.00 | 0.66 | 1.53 | 2.25 | 2.69 | 3.15 | 4.12 | 1.00 |
| $\gamma_{2}$ | 1.09 | 0.00 | 0.30 | 0.59 | 0.87 | 1.05 | 1.27 | 1.77 | 1.00 |
| $\gamma_{3}$ | 1.79 | 0.00 | 0.45 | 1.01 | 1.47 | 1.74 | 2.06 | 2.78 | 1.00 |
| $\gamma_{4}$ | 1.74 | 0.00 | 0.47 | 0.90 | 1.42 | 1.71 | 2.03 | 2.76 | 1.00 |
| $\gamma_{5}$ | 2.18 | 0.00 | 0.56 | 1.30 | 1.78 | 2.11 | 2.52 | 3.46 | 1.00 |
| $\gamma_{6}$ | 1.36 | 0.00 | 0.40 | 0.71 | 1.06 | 1.34 | 1.62 | 2.23 | 1.00 |
| $\gamma_{7}$ | 1.84 | 0.00 | 0.60 | 0.92 | 1.39 | 1.76 | 2.21 | 3.19 | 1.00 |
| $\gamma_{8}$ | 1.80 | 0.00 | 1.03 | 0.41 | 0.66 | 2.02 | 2.60 | 3.60 | 1.90 |
| $\gamma_{9}$ | 0.56 | 0.00 | 0.16 | 0.25 | 0.45 | 0.56 | 0.67 | 0.86 | 1.00 |
| $\gamma_{10}$ | 2.75 | 0.00 | 0.67 | 1.53 | 2.27 | 2.71 | 3.19 | 4.16 | 1.00 |
| $\gamma_{11}$ | 2.59 | 0.00 | 0.63 | 1.47 | 2.15 | 2.56 | 2.99 | 3.91 | 1.00 |
| $\gamma_{12}$ | 2.02 | 0.00 | 0.59 | 1.03 | 1.60 | 1.97 | 2.38 | 3.33 | 1.00 |
| $\gamma_{13}$ | 1.30 | 0.00 | 0.38 | 0.67 | 0.99 | 1.29 | 1.56 | 2.11 | 1.00 |
| $\gamma_{14}$ | 1.29 | 0.00 | 0.35 | 0.65 | 1.06 | 1.28 | 1.51 | 2.03 | 1.00 |
| $\gamma_{15}$ | 3.19 | 0.00 | 0.62 | 2.07 | 2.75 | 3.16 | 3.60 | 4.50 | 1.50 |
| $\gamma_{16}$ | 1.30 | 0.00 | 0.35 | 0.75 | 1.05 | 1.26 | 1.51 | 2.11 | 1.00 |
| ${p_{0}}_{1}$ | 0.55 | 0.00 | 0.11 | 0.35 | 0.48 | 0.55 | 0.63 | 0.78 | 1.00 |
| ${p_{0}}_{2}$ | 0.43 | 0.00 | 0.28 | 0.03 | 0.19 | 0.39 | 0.66 | 0.95 | 1.00 |
| ${p_{0}}_{3}$ | 0.25 | 0.00 | 0.15 | 0.05 | 0.16 | 0.23 | 0.32 | 0.65 | 1.00 |
| ${p_{0}}_{4}$ | 0.21 | 0.00 | 0.17 | 0.03 | 0.11 | 0.16 | 0.24 | 0.75 | 1.00 |
| ${p_{0}}_{5}$ | 0.85 | 0.00 | 0.11 | 0.63 | 0.80 | 0.86 | 0.92 | 0.98 | 1.00 |
| ${p_{0}}_{6}$ | 0.29 | 0.00 | 0.26 | 0.02 | 0.10 | 0.19 | 0.40 | 0.93 | 1.00 |
| ${p_{0}}_{7}$ | 0.34 | 0.00 | 0.17 | 0.09 | 0.24 | 0.31 | 0.41 | 0.79 | 1.00 |
| ${p_{0}}_{8}$ | 0.41 | 0.00 | 0.43 | 0.02 | 0.06 | 0.12 | 0.95 | 1.00 | 2.10 |
| ${p_{0}}_{9}$ | 0.65 | 0.00 | 0.17 | 0.32 | 0.53 | 0.65 | 0.77 | 0.95 | 1.00 |
| ${p_{0}}_{10}$ | 0.49 | 0.00 | 0.10 | 0.29 | 0.42 | 0.49 | 0.56 | 0.70 | 1.00 |
| ${p_{0}}_{11}$ | 0.45 | 0.00 | 0.10 | 0.27 | 0.39 | 0.45 | 0.52 | 0.66 | 1.00 |
| ${p_{0}}_{12}$ | 0.24 | 0.00 | 0.12 | 0.06 | 0.17 | 0.22 | 0.28 | 0.56 | 1.00 |
| ${p_{0}}_{13}$ | 0.30 | 0.00 | 0.28 | 0.01 | 0.08 | 0.17 | 0.47 | 0.94 | 1.00 |
| ${p_{0}}_{14}$ | 0.60 | 0.00 | 0.26 | 0.05 | 0.43 | 0.65 | 0.81 | 0.97 | 1.00 |
| ${p_{0}}_{15}$ | 0.09 | 0.00 | 0.03 | 0.04 | 0.07 | 0.09 | 0.11 | 0.15 | 1.00 |
| ${p_{0}}_{16}$ | 0.38 | 0.00 | 0.24 | 0.04 | 0.19 | 0.32 | 0.52 | 0.91 | 1.00 |

**Appendix C**

Sampling output of the posterior distributions of the hyper-parameters $\mu_{\gamma}$, $\sigma_{\gamma}$, $\mu_{p_{0}}$, $\sigma_{p_{0}}$ and of each individual the parameters $\gamma$, and $p_{0}$, with hierarchical inter-individual differences. $\hat{R}$ is a measure for convergence of different sampling chains (Gelman & Rubin, 1992), at convergence $\hat{R}=1$.

Table C.1

*Posterior distributions of hyper-parameters* $\mu_{\gamma}$*,* $\sigma_{\gamma}$*,* $\varphi$*,* $\lambda$ *and each individual parameter* $\gamma$*, and* $p_{0}$ *for condition* $P_{c}L_{c}$*.*

|  | *M* | *SE* | *SD* | 2.5% | 25% | 50% | 75% | 97.5% | $\hat{R}$ |
| --- | --- | --- | --- | --- | --- | --- | --- | --- | --- |
| $\mu_{\gamma}$ | 0.86 | 0.00 | 0.15 | 0.63 | 0.77 | 0.85 | 0.94 | 1.21 | 1.00 |
| $\sigma_{\gamma}$ | 0.30 | 0.00 | 0.16 | 0.09 | 0.19 | 0.27 | 0.36 | 0.70 | 1.00 |
| $\varphi$ | 0.67 | 0.00 | 0.13 | 0.40 | 0.58 | 0.68 | 0.77 | 0.90 | 1.00 |
| $\lambda$ | 2.69 | 0.02 | 5.20 | 0.29 | 0.75 | 1.33 | 2.64 | 14.20 | 1.00 |
| $\gamma_{1}$ | 0.84 | 0.00 | 0.25 | 0.48 | 0.68 | 0.8 | 0.94 | 1.46 | 1.00 |
| $\gamma_{2}$ | 0.7 | 0.00 | 0.17 | 0.40 | 0.58 | 0.69 | 0.80 | 1.06 | 1.00 |
| $\gamma_{3}$ | 0.97 | 0.00 | 0.32 | 0.55 | 0.76 | 0.91 | 1.10 | 1.79 | 1.00 |
| $\gamma_{4}$ | 0.55 | 0.00 | 0.13 | 0.30 | 0.46 | 0.55 | 0.64 | 0.80 | 1.00 |
| $\gamma_{5}$ | 0.92 | 0.00 | 0.30 | 0.50 | 0.72 | 0.87 | 1.06 | 1.66 | 1.00 |
| $\gamma_{6}$ | 0.48 | 0.00 | 0.12 | 0.27 | 0.40 | 0.47 | 0.56 | 0.73 | 1.00 |
| $\gamma_{7}$ | 0.82 | 0.00 | 0.28 | 0.46 | 0.66 | 0.77 | 0.92 | 1.54 | 1.00 |
| $\gamma_{8}$ | 0.73 | 0.00 | 0.17 | 0.43 | 0.62 | 0.72 | 0.84 | 1.10 | 1.00 |
| $\gamma_{9}$ | 0.96 | 0.00 | 0.33 | 0.52 | 0.74 | 0.89 | 1.10 | 1.78 | 1.00 |
| $\gamma_{10}$ | 1.01 | 0.00 | 0.33 | 0.58 | 0.80 | 0.95 | 1.14 | 1.83 | 1.00 |
| $\gamma_{11}$ | 1.00 | 0.00 | 0.34 | 0.54 | 0.77 | 0.94 | 1.15 | 1.83 | 1.00 |
| $\gamma_{12}$ | 1.01 | 0.00 | 0.33 | 0.57 | 0.79 | 0.94 | 1.14 | 1.83 | 1.00 |
| $\gamma_{13}$ | 0.51 | 0.00 | 0.12 | 0.29 | 0.42 | 0.50 | 0.59 | 0.75 | 1.00 |
| $\gamma_{14}$ | 0.97 | 0.00 | 0.33 | 0.52 | 0.75 | 0.91 | 1.11 | 1.79 | 1.00 |
| $\gamma_{15}$ | 1.01 | 0.00 | 0.33 | 0.56 | 0.79 | 0.95 | 1.14 | 1.82 | 1.00 |
| $\gamma_{16}$ | 0.94 | 0.00 | 0.30 | 0.52 | 0.74 | 0.89 | 1.09 | 1.68 | 1.00 |
| ${p_{0}}_{1}$ | 0.68 | 0.00 | 0.32 | 0.00 | 0.47 | 0.78 | 0.95 | 1.00 | 1.00 |
| ${p_{0}}_{2}$ | 0.74 | 0.00 | 0.26 | 0.06 | 0.59 | 0.83 | 0.96 | 1.00 | 1.00 |
| ${p_{0}}_{3}$ | 0.66 | 0.00 | 0.32 | 0.0039 | 0.43 | 0.76 | 0.94 | 1.00 | 1.00 |
| ${p_{0}}_{4}$ | 0.84 | 0.01 | 0.19 | 0.34 | 0.76 | 0.92 | 0.99 | 1.00 | 1.00 |
| ${p_{0}}_{5}$ | 0.62 | 0.00 | 0.33 | 0.0027 | 0.34 | 0.7 | 0.93 | 1.00 | 1.00 |
| ${p_{0}}_{6}$ | 0.89 | 0.00 | 0.13 | 0.54 | 0.84 | 0.95 | 0.99 | 1.00 | 1.00 |
| ${p_{0}}_{7}$ | 0.73 | 0.00 | 0.31 | 0.00088 | 0.58 | 0.86 | 0.98 | 1.00 | 1.00 |
| ${p_{0}}_{8}$ | 0.68 | 0.00 | 0.29 | 0.04 | 0.49 | 0.76 | 0.93 | 1.00 | 1.00 |
| ${p_{0}}_{9}$ | 0.64 | 0.00 | 0.33 | 0.0021 | 0.39 | 0.74 | 0.94 | 1.00 | 1.00 |
| ${p_{0}}_{10}$ | 0.65 | 0.00 | 0.31 | 0.0069 | 0.42 | 0.74 | 0.93 | 1.00 | 1.00 |
| ${p_{0}}_{11}$ | 0.61 | 0.00 | 0.34 | 0.0016 | 0.33 | 0.71 | 0.93 | 1.00 | 1.00 |
| ${p_{0}}_{12}$ | 0.65 | 0.00 | 0.31 | 0.0073 | 0.42 | 0.75 | 0.93 | 1.00 | 1.00 |
| ${p_{0}}_{13}$ | 0.9 | 0.00 | 0.12 | 0.55 | 0.85 | 0.95 | 0.99 | 1.00 | 1.00 |
| ${p_{0}}_{14}$ | 0.63 | 0.00 | 0.33 | 0.00 | 0.36 | 0.72 | 0.93 | 1.00 | 1.00 |
| ${p_{0}}_{15}$ | 0.63 | 0.00 | 0.32 | 0.00 | 0.37 | 0.72 | 0.92 | 1.00 | 1.00 |
| ${p_{0}}_{16}$ | 0.61 | 0.00 | 0.34 | 0.00 | 0.31 | 0.69 | 0.93 | 1.00 | 1.00 |

Table C.2

*Posterior distributions of hyper-parameters* $\mu_{\gamma}$*,* $\sigma_{\gamma}$*,* $\varphi$*,* $\lambda$ *and each individual parameter* $\gamma$*, and* $p_{0}$ *for condition* $P_{u}L_{c}$*.*

|  | *M* | *SE* | *SD* | 2.5% | 25% | 50% | 75% | 97.5% | $\hat{R}$ |
| --- | --- | --- | --- | --- | --- | --- | --- | --- | --- |
| $\mu_{\gamma}$ | 0.76 | 0.00 | 0.14 | 0.55 | 0.67 | 0.74 | 0.84 | 1.11 | 1.00 |
| $\sigma_{\gamma}$ | 0.31 | 0.00 | 0.15 | 0.11 | 0.21 | 0.28 | 0.38 | 0.69 | 1.00 |
| $\varphi$ | 0.56 | 0.00 | 0.12 | 0.33 | 0.47 | 0.56 | 0.64 | 0.79 | 1.00 |
| $\lambda$ | 2.70 | 0.01 | 3.73 | 0.44 | 1.03 | 1.67 | 2.90 | 11.83 | 1.00 |
| $\gamma_{1}$ | 0.95 | 0.00 | 0.31 | 0.52 | 0.73 | 0.89 | 1.09 | 1.72 | 1.00 |
| $\gamma_{2}$ | 0.94 | 0.00 | 0.28 | 0.53 | 0.75 | 0.90 | 1.09 | 1.61 | 1.00 |
| $\gamma_{3}$ | 0.82 | 0.00 | 0.32 | 0.44 | 0.62 | 0.74 | 0.93 | 1.66 | 1.00 |
| $\gamma_{4}$ | 0.44 | 0.00 | 0.10 | 0.26 | 0.36 | 0.43 | 0.50 | 0.66 | 1.00 |
| $\gamma_{5}$ | 0.55 | 0.00 | 0.12 | 0.32 | 0.46 | 0.54 | 0.63 | 0.81 | 1.00 |
| $\gamma_{6}$ | 0.44 | 0.00 | 0.11 | 0.25 | 0.36 | 0.43 | 0.51 | 0.67 | 1.00 |
| $\gamma_{7}$ | 0.99 | 0.00 | 0.32 | 0.55 | 0.78 | 0.93 | 1.14 | 1.79 | 1.00 |
| $\gamma_{8}$ | 0.73 | 0.00 | 0.18 | 0.42 | 0.60 | 0.71 | 0.84 | 1.14 | 1.00 |
| $\gamma_{9}$ | 0.44 | 0.00 | 0.10 | 0.26 | 0.37 | 0.43 | 0.50 | 0.65 | 1.00 |
| $\gamma_{10}$ | 0.99 | 0.00 | 0.31 | 0.57 | 0.78 | 0.94 | 1.13 | 1.76 | 1.00 |
| $\gamma_{11}$ | 0.64 | 0.00 | 0.16 | 0.37 | 0.53 | 0.63 | 0.73 | 0.98 | 1.00 |
| $\gamma_{12}$ | 0.98 | 0.00 | 0.28 | 0.56 | 0.78 | 0.94 | 1.12 | 1.66 | 1.00 |
| $\gamma_{13}$ | 0.72 | 0.00 | 0.21 | 0.44 | 0.60 | 0.69 | 0.80 | 1.25 | 1.00 |
| $\gamma_{14}$ | 0.95 | 0.00 | 0.31 | 0.53 | 0.74 | 0.89 | 1.09 | 1.71 | 1.00 |
| $\gamma_{15}$ | 0.91 | 0.00 | 0.29 | 0.51 | 0.71 | 0.85 | 1.04 | 1.64 | 1.00 |
| $\gamma_{16}$ | 0.75 | 0.00 | 0.26 | 0.43 | 0.59 | 0.69 | 0.82 | 1.49 | 1.00 |
| ${p_{0}}_{1}$ | 0.54 | 0.00 | 0.31 | 0.01 | 0.28 | 0.56 | 0.81 | 1.00 | 1.00 |
| ${p_{0}}_{2}$ | 0.51 | 0.00 | 0.30 | 0.01 | 0.26 | 0.52 | 0.77 | 1.00 | 1.00 |
| ${p_{0}}_{3}$ | 0.41 | 0.00 | 0.34 | 0.00 | 0.09 | 0.33 | 0.68 | 1.00 | 1.00 |
| ${p_{0}}_{4}$ | 0.52 | 0.00 | 0.20 | 0.14 | 0.38 | 0.52 | 0.67 | 0.90 | 1.00 |
| ${p_{0}}_{5}$ | 0.63 | 0.00 | 0.23 | 0.15 | 0.46 | 0.65 | 0.82 | 0.99 | 1.00 |
| ${p_{0}}_{6}$ | 0.54 | 0.00062 | 0.19 | 0.17 | 0.4 | 0.54 | 0.68 | 0.91 | 1.00 |
| ${p_{0}}_{7}$ | 0.54 | 0.007 | 0.31 | 0.01 | 0.29 | 0.56 | 0.81 | 1.00 | 1.00 |
| ${p_{0}}_{8}$ | 0.53 | 0.00 | 0.29 | 0.02 | 0.30 | 0.54 | 0.77 | 0.99 | 1.00 |
| ${p_{0}}_{9}$ | 0.90 | 0.00 | 0.10 | 0.64 | 0.85 | 0.93 | 0.98 | 1.00 | 1.00 |
| ${p_{0}}_{10}$ | 0.56 | 0.00 | 0.30 | 0.01 | 0.32 | 0.59 | 0.83 | 1.00 | 1.00 |
| ${p_{0}}_{11}$ | 0.56 | 0.00 | 0.26 | 0.05 | 0.37 | 0.58 | 0.77 | 0.98 | 1.00 |
| ${p_{0}}_{12}$ | 0.52 | 0.00 | 0.30 | 0.01 | 0.28 | 0.54 | 0.78 | 1.00 | 1.00 |
| ${p_{0}}_{13}$ | 0.70 | 0.00 | 0.28 | 0.00 | 0.53 | 0.77 | 0.93 | 1.00 | 1.00 |
| ${p_{0}}_{14}$ | 0.56 | 0.00 | 0.31 | 0.0077 | 0.31 | 0.59 | 0.83 | 1.00 | 1.00 |
| ${p_{0}}_{15}$ | 0.57 | 0.00 | 0.31 | 0.00 | 0.33 | 0.61 | 0.85 | 1.00 | 1.00 |
| ${p_{0}}_{16}$ | 0.40 | 0.00 | 0.31 | 0.0014 | 0.12 | 0.34 | 0.62 | 1.00 | 1.00 |

Table C.3

*Posterior distributions of hyper-parameters* $\mu_{\gamma}$*,* $\sigma_{\gamma}$*,* $\varphi$*,* $\lambda$ *and each individual parameter* $\gamma$*, and* $p_{0}$ *for condition* $P_{c}L_{u}$*.*

|  | *M* | *SE* | *SD* | 2.5% | 25% | 50% | 75% | 97.5% | $\hat{R}$ |
| --- | --- | --- | --- | --- | --- | --- | --- | --- | --- |
| $\mu_{\gamma}$ | 1.76 | 0.00 | 0.41 | 1.06 | 1.48 | 1.72 | 2.00 | 2.66 | 1.00 |
| $\sigma_{\gamma}$ | 1.38 | 0.01 | 0.61 | 0.55 | 0.96 | 1.26 | 1.67 | 2.88 | 1.00 |
| $\varphi$ | 0.33 | 0.00 | 0.10 | 0.16 | 0.26 | 0.32 | 0.39 | 0.54 | 1.00 |
| $\lambda$ | 1.96 | 0.00 | 1.26 | 0.56 | 1.13 | 1.65 | 2.42 | 5.26 | 1.00 |
| $\gamma_{1}$ | 3.39 | 0.00 | 1.33 | 1.52 | 2.47 | 3.15 | 4.03 | 6.61 | 1.00 |
| $\gamma_{2}$ | 1.14 | 0.00 | 0.37 | 0.52 | 0.9 | 1.10 | 1.34 | 2.00 | 1.00 |
| $\gamma_{3}$ | 2.48 | 0.00 | 1.02 | 1.11 | 1.77 | 2.27 | 2.95 | 5.03 | 1.00 |
| $\gamma_{4}$ | 1.28 | 0.00 | 0.36 | 0.73 | 1.03 | 1.22 | 1.47 | 2.14 | 1.00 |
| $\gamma_{5}$ | 0.89 | 0.00 | 0.35 | 0.23 | 0.66 | 0.89 | 1.11 | 1.63 | 1.00 |
| $\gamma_{6}$ | 0.82 | 0.00 | 0.44 | 0.29 | 0.51 | 0.64 | 1.22 | 1.77 | 1.00 |
| $\gamma_{7}$ | 3.51 | 0.00 | 1.32 | 1.59 | 2.58 | 3.28 | 4.17 | 6.72 | 1.00 |
| $\gamma_{8}$ | 2.60 | 0.00 | 1.20 | 1.02 | 1.77 | 2.36 | 3.16 | 5.60 | 1.00 |
| $\gamma_{9}$ | 0.50 | 0.00 | 0.17 | 0.17 | 0.39 | 0.50 | 0.61 | 0.80 | 1.00 |
| $\gamma_{10}$ | 1.32 | 0.00 | 0.43 | 0.54 | 1.04 | 1.37 | 1.60 | 2.13 | 1.00 |
| $\gamma_{11}$ | 3.04 | 0.00 | 1.34 | 1.17 | 2.11 | 2.80 | 3.71 | 6.29 | 1.00 |
| $\gamma_{12}$ | 2.71 | 0.00 | 1.00 | 1.39 | 2.01 | 2.50 | 3.18 | 5.26 | 1.00 |
| $\gamma_{13}$ | 0.47 | 0.00 | 0.14 | 0.18 | 0.38 | 0.48 | 0.57 | 0.74 | 1.00 |
| $\gamma_{14}$ | 1.60 | 0.00 | 0.55 | 0.78 | 1.20 | 1.51 | 1.90 | 2.91 | 1.00 |
| $\gamma_{15}$ | 3.31 | 0.00 | 1.34 | 1.35 | 2.38 | 3.08 | 3.99 | 6.56 | 1.00 |
| $\gamma_{16}$ | 1.28 | 0.00 | 0.44 | 0.57 | 0.98 | 1.21 | 1.53 | 2.33 | 1.00 |
| ${p_{0}}_{1}$ | 0.17 | 0.00 | 0.07 | 0.05 | 0.12 | 0.16 | 0.21 | 0.32 | 1.00 |
| ${p_{0}}_{2}$ | 0.32 | 0.00 | 0.26 | 0.00 | 0.10 | 0.26 | 0.48 | 0.92 | 1.00 |
| ${p_{0}}_{3}$ | 0.06 | 0.00 | 0.09 | 0.00 | 0.03 | 0.05 | 0.07 | 0.18 | 1.00 |
| ${p_{0}}_{4}$ | 0.34 | 0.00 | 0.25 | 0.01 | 0.14 | 0.30 | 0.50 | 0.91 | 1.00 |
| ${p_{0}}_{5}$ | 0.26 | 0.00 | 0.27 | 0.00 | 0.05 | 0.16 | 0.41 | 0.94 | 1.00 |
| ${p_{0}}_{6}$ | 0.52 | 0.00 | 0.37 | 0.00 | 0.00 | 0.62 | 0.84 | 1.00 | 1.00 |
| ${p_{0}}_{7}$ | 0.14 | 0.00 | 0.06 | 0.04 | 0.10 | 0.14 | 0.18 | 0.27 | 1.00 |
| ${p_{0}}_{8}$ | 0.49 | 0.00 | 0.22 | 0.08 | 0.33 | 0.49 | 0.66 | 0.92 | 1.00 |
| ${p_{0}}_{9}$ | 0.61 | 0.00 | 0.21 | 0.24 | 0.45 | 0.61 | 0.78 | 0.98 | 1.00 |
| ${p_{0}}_{10}$ | 0.19 | 0.00 | 0.32 | 0.00 | 0.00 | 0.02 | 0.17 | 0.98 | 1.00 |
| ${p_{0}}_{11}$ | 0.19 | 0.00 | 0.11 | 0.04 | 0.12 | 0.17 | 0.24 | 0.47 | 1.00 |
| ${p_{0}}_{12}$ | 0.04 | 0.00 | 0.04 | 0.00 | 0.02 | 0.03 | 0.05 | 0.07 | 1.00 |
| ${p_{0}}_{13}$ | 0.66 | 0.00 | 0.20 | 0.29 | 0.51 | 0.67 | 0.83 | 0.99 | 1.00 |
| ${p_{0}}_{14}$ | 0.18 | 0.00 | 0.20 | 0.0032 | 0.05 | 0.11 | 0.23 | 0.82 | 1.00 |
| ${p_{0}}_{15}$ | 0.19 | 0.00 | 0.09 | 0.06 | 0.13 | 0.18 | 0.23 | 0.39 | 1.00 |
| ${p_{0}}_{16}$ | 0.28 | 0.00 | 0.24 | 0.00 | 0.10 | 0.22 | 0.40 | 0.90 | 1.00 |

Table C.4

*Posterior distributions of hyper-parameters* $\mu_{\gamma}$*,* $\sigma_{\gamma}$*,* $\varphi$*,* $\lambda$ *and each individual parameter* $\gamma$*, and* $p_{0}$ *for condition* $P_{u}L_{u}$*.*

|  | *M* | *SE* | *SD* | 2.5% | 25% | 50% | 75% | 97.5% | $\hat{R}$ |
| --- | --- | --- | --- | --- | --- | --- | --- | --- | --- |
| $\mu_{\gamma}$ | 2.32 | 0.00 | 0.45 | 1.48 | 2.03 | 2.30 | 2.59 | 3.25 | 1.00 |
| $\sigma_{\gamma}$ | 1.66 | 0.00 | 0.66 | 0.78 | 1.21 | 1.53 | 1.96 | 3.30 | 1.00 |
| $\varphi$ | 0.36 | 0.00 | 0.07 | 0.23 | 0.31 | 0.36 | 0.41 | 0.51 | 1.00 |
| $\lambda$ | 3.38 | 0.00 | 1.69 | 1.19 | 2.20 | 3.04 | 4.18 | 7.59 | 1.00 |
| $\gamma_{1}$ | 4.23 | 0.00 | 1.46 | 2.12 | 3.22 | 3.98 | 4.95 | 7.76 | 1.00 |
| $\gamma_{2}$ | 1.23 | 0.00 | 0.34 | 0.65 | 0.99 | 1.20 | 1.43 | 1.99 | 1.00 |
| $\gamma_{3}$ | 2.10 | 0.00 | 0.59 | 1.18 | 1.68 | 2.02 | 2.43 | 3.46 | 1.00 |
| $\gamma_{4}$ | 2.09 | 0.00 | 0.62 | 1.12 | 1.65 | 2.01 | 2.44 | 3.52 | 1.00 |
| $\gamma_{5}$ | 3.24 | 0.00 | 1.25 | 1.48 | 2.38 | 3.02 | 3.86 | 6.31 | 1.00 |
| $\gamma_{6}$ | 1.58 | 0.00 | 0.45 | 0.81 | 1.28 | 1.54 | 1.85 | 2.57 | 1.00 |
| $\gamma_{7}$ | 2.76 | 0.00 | 1.17 | 1.15 | 1.93 | 2.54 | 3.34 | 5.65 | 1.00 |
| $\gamma_{8}$ | 3.54 | 0.00 | 1.21 | 1.84 | 2.69 | 3.32 | 4.14 | 6.55 | 1.00 |
| $\gamma_{9}$ | 0.55 | 0.00 | 0.17 | 0.23 | 0.43 | 0.54 | 0.66 | 0.91 | 1.00 |
| $\gamma_{10}$ | 4.22 | 0.00 | 1.45 | 2.11 | 3.23 | 3.99 | 4.96 | 7.68 | 1.00 |
| $\gamma_{11}$ | 3.74 | 0.00 | 1.22 | 1.94 | 2.90 | 3.55 | 4.35 | 6.66 | 1.00 |
| $\gamma_{12}$ | 2.94 | 0.00 | 1.17 | 1.33 | 2.13 | 2.72 | 3.51 | 5.83 | 1.00 |
| $\gamma_{13}$ | 1.50 | 0.00 | 0.41 | 0.76 | 1.23 | 1.48 | 1.75 | 2.38 | 1.00 |
| $\gamma_{14}$ | 1.34 | 0.00 | 0.43 | 0.63 | 1.02 | 1.31 | 1.61 | 2.26 | 1.00 |
| $\gamma_{15}$ | 4.67 | 0.00 | 1.44 | 2.61 | 3.68 | 4.43 | 5.38 | 8.14 | 1.00 |
| $\gamma_{16}$ | 1.48 | 0.00 | 0.42 | 0.82 | 1.17 | 1.42 | 1.72 | 2.44 | 1.00 |
| ${p_{0}}_{1}$ | 0.51 | 0.00 | 0.09 | 0.36 | 0.45 | 0.51 | 0.58 | 0.69 | 1.00 |
| ${p_{0}}_{2}$ | 0.30 | 0.00 | 0.22 | 0.02 | 0.13 | 0.25 | 0.42 | 0.84 | 1.00 |
| ${p_{0}}_{3}$ | 0.24 | 0.00 | 0.11 | 0.06 | 0.17 | 0.23 | 0.30 | 0.47 | 1.00 |
| ${p_{0}}_{4}$ | 0.18 | 0.00 | 0.10 | 0.03 | 0.12 | 0.17 | 0.22 | 0.41 | 1.00 |
| ${p_{0}}_{5}$ | 0.78 | 0.00 | 0.10 | 0.59 | 0.74 | 0.79 | 0.84 | 0.94 | 1.00 |
| ${p_{0}}_{6}$ | 0.20 | 0.00 | 0.16 | 0.02 | 0.09 | 0.15 | 0.24 | 0.70 | 1.00 |
| ${p_{0}}_{7}$ | 0.30 | 0.00 | 0.10 | 0.12 | 0.25 | 0.30 | 0.34 | 0.53 | 1.00 |
| ${p_{0}}_{8}$ | 0.12 | 0.00 | 0.05 | 0.02 | 0.08 | 0.12 | 0.16 | 0.22 | 1.00 |
| ${p_{0}}_{9}$ | 0.58 | 0.00 | 0.17 | 0.24 | 0.47 | 0.57 | 0.69 | 0.90 | 1.00 |
| ${p_{0}}_{10}$ | 0.48 | 0.00 | 0.09 | 0.32 | 0.42 | 0.48 | 0.54 | 0.65 | 1.00 |
| ${p_{0}}_{11}$ | 0.43 | 0.00 | 0.08 | 0.29 | 0.37 | 0.42 | 0.48 | 0.60 | 1.00 |
| ${p_{0}}_{12}$ | 0.24 | 0.00 | 0.07 | 0.10 | 0.20 | 0.24 | 0.28 | 0.37 | 1.00 |
| ${p_{0}}_{13}$ | 0.18 | 0.00 | 0.18 | 0.01 | 0.07 | 0.13 | 0.22 | 0.74 | 1.00 |
| ${p_{0}}_{14}$ | 0.50 | 0.00 | 0.25 | 0.03 | 0.31 | 0.53 | 0.69 | 0.91 | 1.00 |
| ${p_{0}}_{15}$ | 0.11 | 0.00 | 0.03 | 0.06 | 0.09 | 0.11 | 0.13 | 0.19 | 1.00 |
| ${p_{0}}_{16}$ | 0.29 | 0.00 | 0.18 | 0.03 | 0.17 | 0.26 | 0.38 | 0.75 | 1.00 |

**Appendix D**

Linear Mixed Effect Model for parameters $\gamma$ and $p_{0}$ from the model with full inter-individual differences. Parameters $\gamma$ and $p_{0}$ are modelled through

$$\gamma_{i}=\alpha+\beta_{1}\cdot{prior}_{u|c}+\beta_{2}\cdot{likelihood}_{u|c}+\beta_{3}\cdot{prior}_{u|c}\cdot{likelihood}_{u|c}+{RE}_{subj i}$$

Where $\alpha$ is an intercept term corresponding to the $P_{c}L_{c}$ condition, ${prior}_{u|c}$ is a binary indicator of an uncertain prior condition, ${likelihood}_{u|c}$ is a binary indicator of an uncertain likelihood condition and ${RE}_{subj i}$ is a subject specific intercept. In this model the $\beta$ coefficients indicate the change in the subject-specific $\gamma$ or $p_{0}$ parameters if the prior is uncertain $(\beta_{1})$, the likelihood is uncertain ($\beta_{2})$ or the interaction if both prior and likelihood are uncertain $(\beta_{3})$.

Table D.1

*Linear Mixed Effects Model statistics for parameter* $\gamma$***.***

|  | *Coef.* | *SE* | *z* | p |
| --- | --- | --- | --- | --- |
| $\alpha$ | 1.17 | 0.16 | 7.36 | <0.001 |
| $\beta_{1}$ | -0.11 | 0.17 | -0.66 | 0.512 |
| $\beta_{2}$ | 0.35 | 0.17 | 2.01 | 0.036 |
| $\beta_{3}$ | 0.44 | 0.24 | 1.87 | 0.062 |

Table D.2

*Linear Mixed Effects Model statistics for parameter* $p_{0}$***.***

|  | *Coef.* | *SE* | *z* | p |
| --- | --- | --- | --- | --- |
| $\alpha$ | 0.55 | 0.04 | 13.42 | <0.001 |
| $\beta_{1}$ | -0.02 | 0.05 | -0.35 | 0.730 |
| $\beta_{2}$ | -0.15 | 0.05 | -3.00 | 0.003 |
| $\beta_{3}$ | 0.03 | 0.07 | 0.43 | 0.663 |
